# A male-transmitted B chromosome undergoes strong meiotic drag in females of the jewel wasp *Nasonia vitripennis*

**DOI:** 10.1371/journal.pbio.3003599

**Published:** 2026-01-16

**Authors:** Patrick M. Ferree, Jayla Cummings, Emma Garman, Jacqueline Solomon, Kassandra Soriano Martinez

**Affiliations:** The Department of Natural Sciences, Pitzer College and Scripps College, Claremont, California, United States of America; Whitehead Institute for Biomedical Research, UNITED STATES OF AMERICA

## Abstract

Many organisms carry extra, non-essential chromosomes known as B chromosomes (Bs), which are selfishly transmitted at super-mendelian levels to offspring. This heightened transmission, termed drive, occurs during gametogenesis, usually in one of the two parents. In some cases, Bs can experience an opposing process, drag, which reduces their transmission. If these processes occur together in the same organism, one in each parental sex, then they may facilitate the spread of Bs while countering their accumulation in the genome to harmful levels. While previous studies have elucidated mechanistic aspects of B drive, little is known about drag or other factors that govern the inheritance of these selfish genetic elements. Here, we examined the inheritance of Paternal Sex Ratio (PSR), a single-copy B in the jewel wasp, *Nasonia vitripennis*, which is transmitted paternally to offspring. PSR drives by converting female-destined embryos into PSR-transmitting males. Using genetic manipulation, we produced exceptional PSR-carrying females, which were used to assess B transmission potential. We found that females transmit PSR at an unexpectedly low level compared to univalent chromosomes in other organisms. This reduced transmission stems from remarkable loss of PSR from the egg’s nucleus upon entry into meiosis, an effect that may be caused by an absence of microtubule-based spindle fibers in meiosis I-arrested wasp eggs. We also found that PSR is strictly limited to a single copy per genome, likely because wasps having two PSR copies die during development. Our findings reveal the successful inheritance of this selfish B chromosome involves its restriction to a single copy and hidden female meiotic drag in addition to strong paternal drive.

## Abstract 2

Many organisms carry non-essential, selfish B chromosomes that spread through super-Mendelian transmission, but opposing forces like drag can limit their accumulation. This study shows that the Paternal Sex Ratio (PSR) chromosome in the jewel wasp achieves successful inheritance through strong paternal drive, hidden female meiotic drag, and restriction to a single copy.

## Introduction

Mendelian inheritance predicts the fair transmission of differing alleles from a heterozygous parent to progeny. This fundamental pattern of inheritance relies on equal segregation of homologous chromosomes into sperm or egg during gametogenesis. However, thousands of plants, animals, and fungi carry non-essential chromosomes known as B chromosomes (Bs), many of which exhibit deviant transmission characteristics that are self-serving and, thus, evolutionarily advantageous for these selfish genetic elements [1–3]. The number of Bs typically ranges from 2 to 6 copies per species, but some can reach as high as 30–50 copies [3,4], and within a given species, B copy number can vary greatly among individuals [5]. These peculiar dynamics and, more broadly, the long-term persistence of B chromosomes, are strongly influenced by two opposing phenomena known as *drive* and *drag* [6]. While Bs normally are transmitted to progeny by both parents, they often exhibit super-mendelian (*i.e.*, positively biased) transmission from one or the other parental sex. In many organisms, this biased transmission, known as drive, occurs during female meiosis and is influenced by certain properties of meiotic cell division. For example, in certain organisms such as the mottled grasshopper and the slim-stem lily, Bs segregate more frequently into the egg-destined meiotic product because they tend to aggregate on the longer side of an asymmetrically shaped spindle apparatus during the first meiotic division [7, 8]. Bs in the fruit fly *Drosophila melanogaster* also undergo drive during female meiosis [9], but presumably through a different mechanism because the first meiotic spindle in this organism is not spatially asymmetrical [10,11]. Regardless, these Bs are preferentially transmitted, resulting in progeny possessing as many as 12 B copies per individual [9]. In contrast, in this same insect, Bs are transmitted by the male parent at lower-than-mendelian levels [9] through a process referred to as drag [12]. In such cases where drive and drag occur in the same organism, one in each parental sex, these processes may help to maintain B copy number at levels that prevent B loss and simultaneously prevent B copy number from reaching levels that are deleterious to the organism. It is universally likely that specific aspects of gametogenesis that differ between the sexes lead to drive in females and drag in males in species like *D. melanogaster*, or vice versa in other organisms, and the interplay of drive, drag, and perhaps other unknown factors governs the overall success of B inheritance. While recent studies have helped elucidate mechanistic aspects of B drive in a number of organisms including *D. melanogaster* [9], rye [13], and maize [14], very little is known about the cellular features that underlie drag or otherwise delimit B transmission.

The jewel wasp, *Nasonia vitripennis*, carries a selfish B chromosome known as Paternal Sex Ratio (PSR), which exhibits exceptional but incompletely understood transmission characteristics. PSR is present in as much as 11% frequency in certain wild populations of the Great Salt Lake area of Utah, USA [15]. When present, PSR is found only in the male sex, and it invariantly exists in single copy per genome [16]. Because PSR is only in males, it is transmitted paternally to offspring via the sperm. PSR’s drive involves a *trans-*acting effect that causes elimination of the sperm’s standard genome, but not PSR itself, during the first mitotic division following fertilization [17, 18]. The reproductive mode of *N. vitripennis* and all other hymenopteran insects (including all wasps, bees, and ants) is haplo-diploidy, whereby males develop as haploids from unfertilized eggs, which have a single set of essential chromosomes, while females develop as diploids from fertilized eggs, having two sets of essential chromosomes. The intrinsic fertilization rate of *N. vitripennis* is 80%–90%, which normally leads to broods of progeny that are proportionally female-biased. However, when PSR is present, the sperm’s half of the genome is eliminated. As a result, female-destined embryos are converted into PSR-carrying (PSR+), and thus transmitting, males. This paternal genome elimination (PGE) event and the resulting female-to-male conversion are highly effective, occurring in over 99% of eggs fertilized by PSR+ sperm [19]. Due to the strength of this effect, and given the 80%–90% fertilization rate, the result is the production of all-male broods, with the majority of males being PSR+. Due to the harsh effects of PSR on the wasp’s genome and sex ratio, this B has been referred to as the most extreme selfish genetic element currently known [20].

The exceptional inheritance characteristics of PSR beg several important questions. First, why is the transmission of this B restricted to the male sex? Part of the answer is likely that PSR’s strong female-to-male conversion effect leads to this B invariantly being in the male sex. It is formally possible that PSR could be transmitted maternally, or even undergo meiotic drive like other Bs, if it were to find itself in females. Alternatively, there may be unknown fitness costs to females that carry PSR, or intrinsic cellular barriers to PSR transmission during female gametogenesis. It is noteworthy that unlike haploid male wasps, which produce sperm entirely through mitotic division [21], egg production in diploid females involves standard meiosis [22]. This difference between the sexes opens the possibility that PSR may encounter different cellular influences when transmitted by each parent. A second important question is: why is PSR present only as a single copy in the wasp genome? This characteristic may result from a lack of opportunity for PSR to reach a higher copy number because it is not transmitted by both parents. Instead, there may be a genetic constraint to the B being present in two or more copies per genome.

We tested these ideas by experimentation with artificially produced, PSR+ females. Previously, it was shown that expression of a PSR gene named *haploidizer* is required for PSR’s genome elimination activity [23]. When transcripts of this gene are targeted for degradation by systemic RNAi (sRNAi), PSR’s genome-eliminating activity is suppressed, leading to the production of daughters that carry PSR [23]. Using these individuals, we found that PSR does not affect female fitness, and females are capable of transmitting PSR to F1 progeny. However, the level of maternal transmission is much lower than predicted for a univalent chromosome, compared to univalent segregation in *D. melanogaster*. This transmission reduction stems from PSR being lost from the maternal nucleus upon entry into the first meiotic division, before chromosome segregation. Thus, PSR experiences meiotic drag in females, which is a rare occurrence due to PSR’s strong female-to-male conversion activity that enables its transmission through males. Finally, crosses between PSR+ males and females produced numerous adult offspring with a single PSR copy but none with two PSR copies. A portion of these offspring arrested during late larval development, suggesting that more than one copy of PSR is harmful. Together, our findings argue that the successful inheritance of PSR depends largely on the balance between its strong paternal drive and restriction to one copy, although the observed maternal drag, normally masked by strong drive, may help eliminate non-driving PSR variants and ultimately enhance wasp fitness.

## Results

### PSR undergoes normal mitotic segregation in females and does not affect their fitness or fecundity

**Fig 1:**
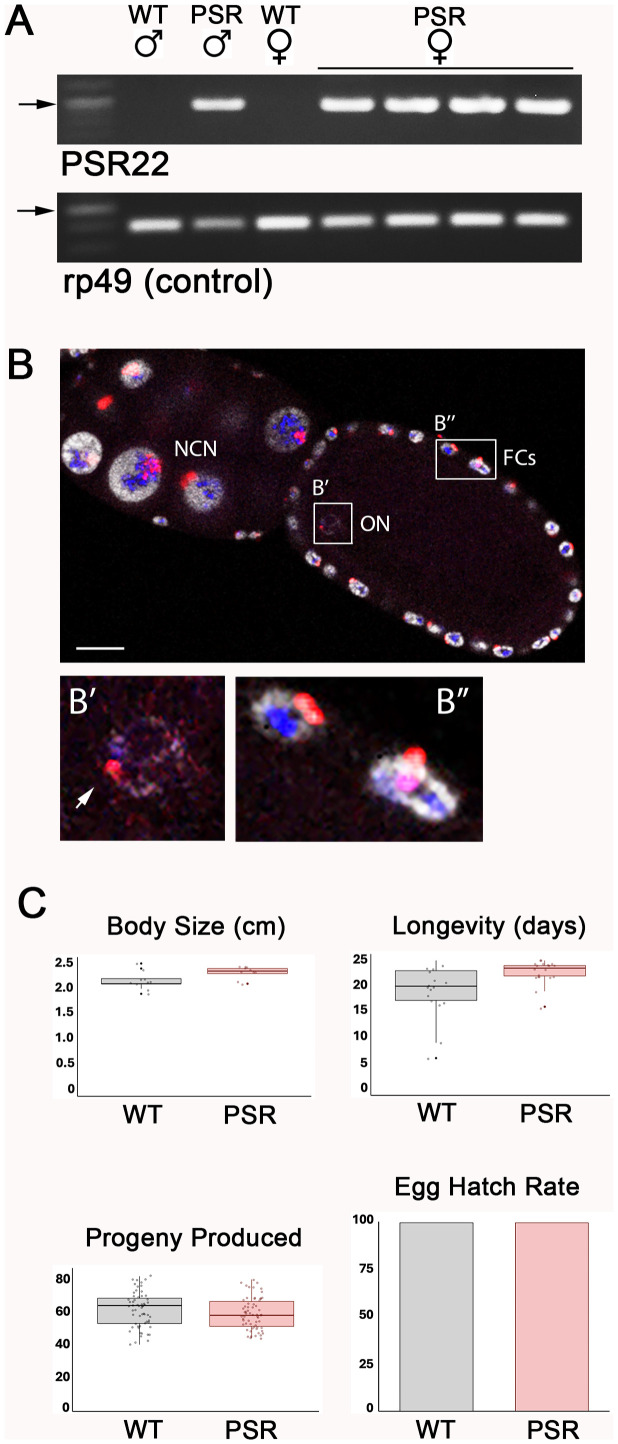
Genetically produced PSR+ females are similarly fit to wildtype females. **(A)** An agarose genotyping gel showing amplification of a ~1 kilobase pair PSR-specific PCR product from the genomic DNA of F1 females produced by PSR+ fathers that were sRNAi-treated for degradation of *haploidizer* transcripts. The housekeeping gene, rp49 is used as an amplification control. The arrows indicate a 1,000nt band in the size standard ladder. The original gel images can be found in S1 Data. **(B)** A mid-stage egg chamber taken from a PSR+ female, when hybridized with a PSR probe cognate to the *haploidizer* gene, reveals the presence of PSR (red) in the polyploid nurse cell nuclei (NCN), the oocyte nucleus (ON) (**B′**), and somatic follicle cells (FCs) (**B**″). rDNA is shown in blue, and DNA is gray. Scale bar equals 15 μM. (**B′**) and (**B**″) are shown in higher magnification. The white arrow in (**B′**) indicates the single copy of PSR in the chromatin body of the germinal vesicle. **(C)** Four different characteristics of fitness—body size, longevity, progeny produced per PSR+ female, and hatch rate for eggs laid by unmated PSR+ females—are shown in comparison with the same characteristics for wild-type females. Averages and standard deviations are shown for the first three characteristics. Supporting data for these graphs can be found in S2 Data.

Previously, it was determined that genetically produced PSR+ females do not transmit PSR to their offspring [23]. To revisit this finding, we generated PSR+ females using the same approach (see Materials and methods). After confirming the PSR-carrying status of these individuals with PCR (Fig 1A and S1 Data), we microscopically examined their ovaries. Like wild-type ovaries, PSR+ ovaries contained developing oocytes that were morphologically normal (Fig 1B). Using DNA fluorescence in situ hybridization (FISH), we visualized PSR with a probe that is cognate to a high-copy satellite sequence uniquely located on PSR; this probe labels both arms of the B chromosome [24]. PSR was present in the nuclei of nurse cells in early-, mid-, and late-stage egg chambers (Fig 1B). In these nuclei, PSR was amplified to high copy number, as indicated by the large region of FISH signal, like the wasp’s rDNA locus (Fig 1B). This pattern reflects the normal polyploidization of DNA in these cells [25]. PSR also appeared in the nuclei of the somatic follicle cells that surround each egg chamber, and as a small, single focus inside the oocyte’s germinal vesicle (Fig 1B). We saw no nuclei in the ovary that were devoid of PSR, indicating that PSR segregates effectively during the numerous mitotic divisions giving rise to the female’s germline and somatic cells. Additionally, PSR+ females were like wild type females in body size, longevity, egg production, and number of offspring produced per female (Fig 1C and S2 Data). Thus, PSR has no measurable effect on female fitness or fecundity, and PSR+ females do not seem to suffer from potential aneuploidy or other chromosome abnormalities that may arise from the imperfect PGE suppression that was previously observed to occur in some young embryos because of the sRNAi treatment used to produce them [23].

### Females transmit PSR to progeny at lower-than-expected levels for a univalent chromosome

**Fig 2:**
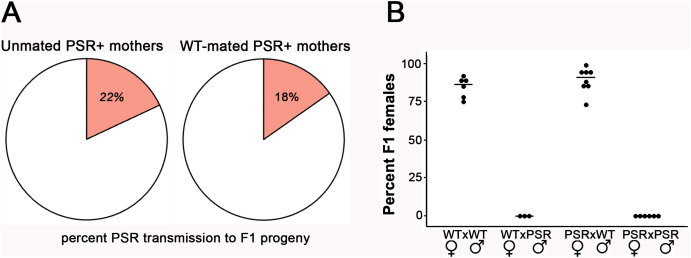
Mating status does not influence the low maternal transmission of PSR. **(A)** The percentage of F1 progeny inheriting PSR from unmated PSR+ mothers (left pie chart) or PSR+ mothers mated with wildtype males (right chart). The transmission levels for these conditions are very similar. Supporting data can be found in S3 Data. **(B)** Maternally transmitted PSR does not cause PGE and sex ratio distortion, nor does it affect PGE caused by paternally transmitted PSR. Sex ratio values for adult F1 broods produced by PSR+ mothers are shown on the y-axis. The female × male genotypes are shown for each cross on the x-axis. The crosses between wild type males and females and between wild-type females crossed with PSR+ males serve as controls—the percent females in their F1 progeny are 80%–90% and 0%, respectively. Supporting data can be found in S4 Data.

To assess the maternal transmission of PSR, we used PCR to genotype broods of all-male F1 offspring produced by unmated, PSR+ females. Previous genetic and microscopic studies of univalent segregation in *D. melanogaster* were considered to estimate an expected level of PSR transmission from mothers. In the fruit fly, univalent chromosomes derived from fusions of two X chromosomes, two fourth chromosomes, or an arm from each of chromosomes 2 and 3 exhibited no substantial segregation defects during meiosis and were transmitted effectively to an estimated half of all eggs, when accounting for lethal progeny that do not receive a homologous counterpart [26]. In contrast to these univalent chromosomes in the fruit fly, PSR was detected in only 22 percent of offspring laid by these unmated female wasps (Fig 2A and S3 Data). The transmission level was similar when PSR+ females were crossed with wild-type males (Fig 2A), showing that fertilization does not affect the level of PSR transmission by females. These findings argue that, even though PSR segregates properly during mitosis in females and is present in the germinal vesicle of developing oocytes, this B is transmitted at a much lower level than previously observed for univalent chromosomes in the fruit fly. Notably, PSR+ females when crossed with PSR+ males produced all-male broods, like wild type females crossed with PSR+ males (Fig 2B and S4 Data). This finding demonstrates that the presence of PSR in the egg does not impact the PGE-inducing activity of the paternally transmitted PSR copy.

### PSR becomes lost from the nucleus upon entry into meiosis

**Fig 3:**
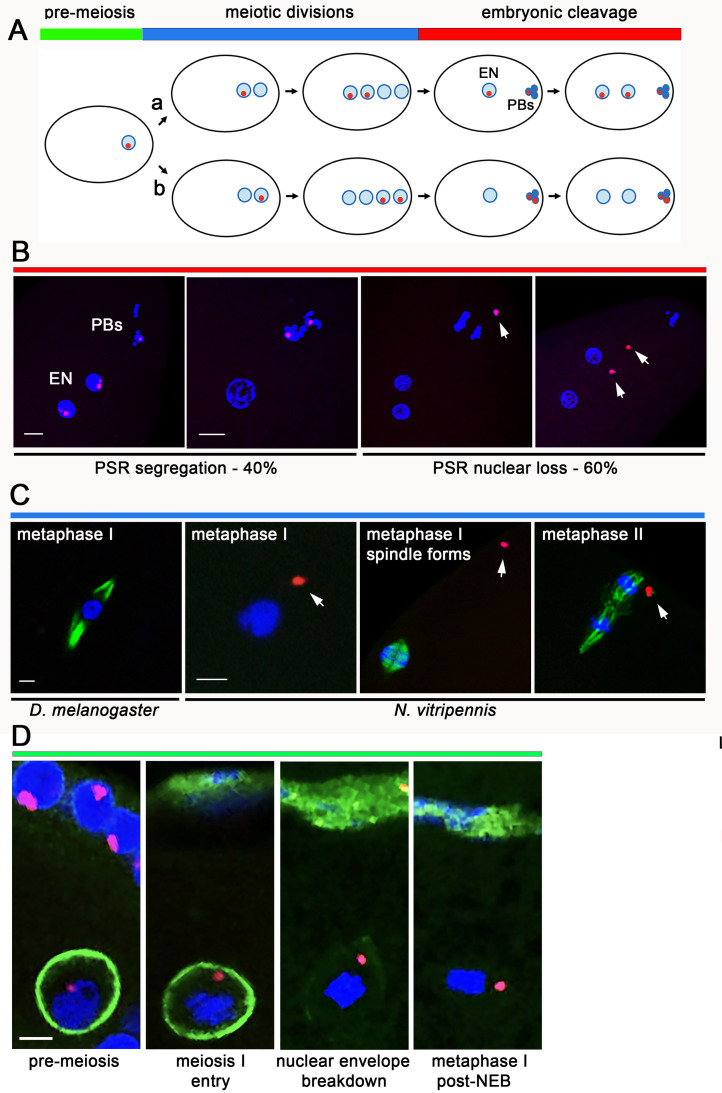
PSR is lost from the egg’s nuclear material upon entry into meiosis. (**A**) schematic of PSR segregation during entry into meiosis, the meiotic divisions, and the first embryonic mitotic division. Under this model, PSR is depicted to segregate normally to two of four meiotic products if there is a functional distributive system that ensures proper segregation of achiasmatic and univalent chromosomes. Two segregation scenarios are shown: (a) the sister PSR chromatid pair moves to the inner product at the end of meiosis I, leading to PSR being present in the egg’s nucleus and one polar body. In (b), the sister PSR chromatid pair moves to the outer meiotic product during meiosis I; in this case, PSR would not be inherited. Details of PSR segregation during metaphase of meiosis I and II are shown in S5 Data. **(B)** The first embryonic mitotic division is shown in the panels, with different outcomes of PSR segregation. From left to right: PSR segregates to the two mitotic daughters and one polar body (*i.e.*, the scenario (a) above); PSR (red) segregates to two of the three polar bodies (*i.e*., the scenario (b) above); loss of the sister PSR chromatid pair from the polar bodies; and loss of the PSR chromatids separately from the nuclei. Scale bars in the left panels equal 10 μM. White arrows indicate the PSR sister pair or individual chromatids. Polar bodies are labeled as PBs, and the embryonic nucleus is EN. Supporting data images can be found in S6 Data. **(C)** Panels show meiotic nuclei at different stages. The left-most panel shows the egg’s nuclear material arrested in meiosis 1 in a pre-activated *Drosophila melanogaster* egg (control); a bipolar spindle apparatus (green) is clearly visible. The next three panels are meiotic nuclei in *N. vitripennis* eggs. From left to right: Meiosis I arrest in a pre-activated, mature egg, in which the chromatin is condensed but spindle fibers are not visible. PSR has already become lost from the meiotic nucleus by this time; Metaphase of meiosis I in a newly activated egg, in which the spindle fibers (green) are apparent and PSR has drifted afar from the spindle; Metaphase of meiosis II in a slightly older, activated egg, showing a tripolar spindle and PSR at an adjacent position. White arrows indicate PSR. Scale bars equal 8 μM. **(D)** From left to right, panels depict the nuclear envelope of the germinal vesicle (green) in an immature egg, in which PSR is still associated with the chromatin body; the nuclear envelope begins to break down upon entry into meiosis I, and PSR becomes displaced from the condensed chromatin within the nucleoplasm; nuclear envelope breakdown (NEB) is nearly complete; NEB has finished. Scale bar equals 10 μM.

To gain insight into the low maternal transmission of PSR, we microscopically inspected 1–2 hours unfertilized embryos laid by unmated PSR+ females. At this time, the meiotic divisions within the egg’s cytoplasm have completed, and one of the four meiotic products which becomes the haploid embryonic nucleus has initiated syncytial mitotic divisions (Fig 3A and S5 Data). The other three meiotic products have become hyper-condensed polar bodies that cluster at a position near the plasma membrane and do not undergo division (Fig 3A and S5 Data). In 40% percent of these embryos (*n* = 53/133), the two sister PSR chromatids occupied either two polar bodies or one polar body and the mitotic cleavage nuclei, reflecting normal segregation patterns (Fig 3A and 3B and S6 Data). However, in the remaining 60 percent of embryos (*n* = 80/133), either one or both sister PSR chromatids were found outside the mitotic nuclei or polar bodies, sometimes at a great distance from them in the cytoplasm (Fig 3B and S6 Data).

To identify when PSR becomes lost from the nuclei, we examined eggs undergoing meiosis. For comparison, the hereditary material of mature, pre-activated *D. melanogaster* eggs is arrested at metaphase of meiosis I [27]. Initially, we examined mature fly eggs to confirm that our experimental conditions were conducive to visualizing the spindle apparatus. In these cells, a well-formed bipolar spindle could be clearly seen to emanate from the body of condensed meiotic chromosomes under fluorescent microscopy using an antibody against a-Tubulin (Fig 3C). In contrast, in mature, pre-activated *N. vitripennis* eggs, the hereditary material was condensed but the spindle was not yet visible (*n* = 7/7; Fig 3C). By this time, PSR has become detached from the nucleus (Fig 3C). Given our prior observation that PSR is present within the chromatin of the germinal vesicle in immature eggs before the initiation of meiosis (Fig 1B), it stands to reason that PSR becomes lost from the nucleus upon entry into meiosis I. The displaced PSR chromatids were also observed at metaphase of the first and second meiotic divisions, when the spindle apparatus has clearly formed (Fig 3C). In contrast, the wasp’s essential chromosomes remained within the nuclei during all steps of meiosis and the early embryonic mitotic divisions (Fig 3C). Thus, the chromosomal loss is specific to PSR. Finally, we wondered if PSR loss from the egg’s nucleus may result from PSR being excluded outside the nuclear envelope. Visualization of the nuclear envelope with an anti-Lamin antibody showed that this was not the case; PSR became displaced from the chromatin body and into the nucleoplasm before nuclear envelope breakdown (Fig 3D).

### PSR copy number is constrained to one per genome

**Fig 4:**
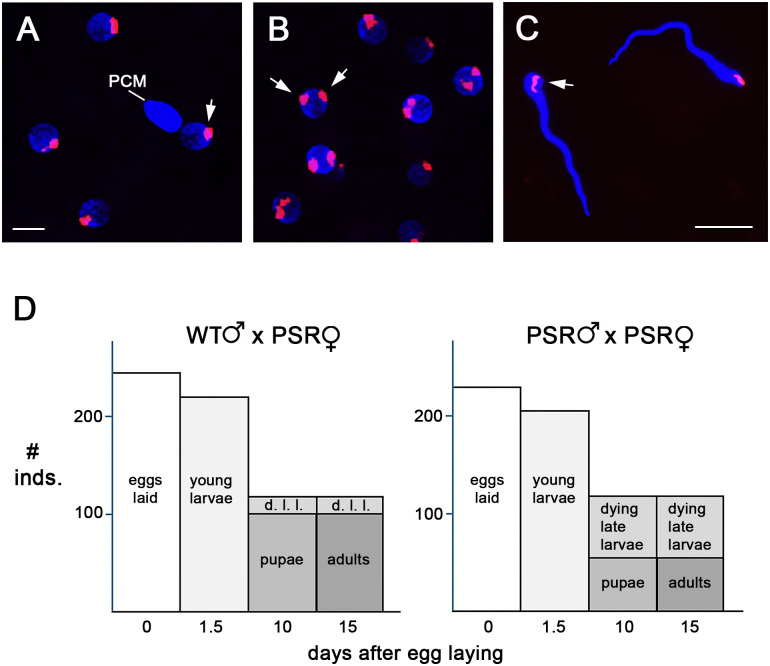
PSR is restricted to single-copy status in adult F1 males produced by PSR+ parents. **(A)** Nuclei in a 1–2-hour-old embryo produced by PSR+ parents. Each nucleus contains a single focus of PSR. **(B)** Nuclei in a slightly older embryo produced by the same parents as **(A)**. Each nucleus contains two well-separated PSR foci (white arrows). Supporting data for embryo scorings can be found in S7 Data. **(C)** Nuclei of mature sperm cells from F1 adults produced by the same parents; each contains a single PSR focus (white arrow). Scale bars in (A) and (C) are 10 μM and 5 μM, respectively. Supporting data for embryo scorings can be found in S8 and S9 Data. **(D)** Graphs showing the developmental advancement of progeny from wild-type fathers and PSR+ mothers (left) and progeny from PSR+ fathers and PSR+ mothers (right). Supporting data used to generate these graphs can be found in S10 Data.

Given that PSR is invariably present in single copy per wasp genome in natural populations and laboratory stocks, we tested if wasps could carry two PSR copies. To do this, we crossed the genetically produced PSR+ females with PSR+ males and microscopically examined B copy number in their F1 progeny. Considering the measured maternal transmission rate of 22 percent, the fact that effectively all sperm of PSR+ males carry PSR [28], and >80% of eggs are fertilized [29], we estimated that 17.6 percent of F1 progeny from these crosses should carry two copies of PSR. We found that 16.7 percent of young F1 embryos (*n* = 13/78) contained two clearly distinct and spatially separated PSR foci, one copy from each gamete, in each mitotic nucleus (Fig 4A and 4B and S7 Data). The remainder of F1 embryos (*n* = 65/78) contained a single PSR focus in each nucleus, likely reflecting the copy delivered by the sperm (Fig 4A and 4B and S7 Data). We then assessed PSR copy number in F1 adults by examining the nuclei of their individualized, mature sperm cells. In all examined sperm from numerous males generated by different cross replicates (*n* = 171/171 males from 7 different PSR+ × PSR+ crosses), we observed only a single PSR copy (Fig 4C and S8 Data). Given that sperm chromatin is packaged into an extraordinarily condensed state [30], it is possible that we may have misassigned sperm that appear to contain a single PSR copy when instead they contain two PSR copies that localize closely together, appearing as one. To address this possibility, we crossed F1 males produced by PSR+ parents with wild-type females and examined the nuclei of their progeny during early embryogenesis, when we are capable of clearly distinguishing between one or two PSR copies. We observed no embryos from these crosses in which there were two PSR copies per nucleus (*n* = 0/57; S9 Data). Instead, all these embryos contained one PSR focus per nucleus. Thus, there appears to be a strict limitation of PSR’s copy number to one.

We speculated that the absence of adults having two PSR copies may result from failure of these individuals to survive to adulthood. To test this hypothesis, we followed the development of F1 progeny produced by PSR+ parents. For comparison, we also examined F1 progeny from wild-type females crossed with PSR+ males. For both conditions, there was a substantial loss of progeny between the first and third larval stages (Fig 4D and S10 Data). This loss likely stems from proneness of the young larvae to desiccation when the host’s shell is opened to score progeny numbers (see Materials and methods). The developmental profiles of these two conditions were nearly identical with one exception: only 46% of progeny produced by PSR+ parents successfully advanced from the late larval to the pupal stage, whereas 87% of control progeny made this transition (Fig 4D and S10 Data). While about half of the larvae from PSR+ parents were still alive albeit with arrested development, potentially indicating diapause, the other half stopped moving and turned dark, indicating early death. It stands to reason that a portion of these individuals carry two PSR copies. These results suggest that the larval-to-pupal transition is sensitive to this genotype.

## Discussion

In this study, we investigated two unexplored aspects of PSR inheritance by utilizing genetically produced PSR+ females. The first aspect is whether female wasps can transmit PSR. Normally, this B chromosome is carried only by males due to its strong female-to-male-converting activity through PGE. Thus, the female sex is bypassed under normal circumstances. In a previous study, shearing the paternal genome with genetic manipulation produced rare females carrying PSR chromosomal variants [31]. These PSR variants possessed deleted regions, possibly including the locus responsible for PGE activity. These PSR variants were transmitted from females to progeny at low levels, but it was unclear if this effect reflected an intrinsically low female transmission or, instead, it was due to instability caused by the PSR deletions. Our study involved analyses of the intact PSR chromosome placed into females by sRNAi-targeting the PGE-causing locus, thereby circumventing any confounding effects of abnormal segregation due to chromosomal abnormalities. Here, we found that, just as there are no observable fitness differences between PSR+ and wild-type males [32], the same is true for females. A previous study using the same sRNAi approach observed a complete lack of PSR transmission by females [23]. We speculate that this effect may have arisen from certain PCR conditions that were used for genotyping. Here, we unambiguously demonstrated with PCR and microscopic analysis that PSR+ females are indeed capable of transmitting PSR to progeny. However, PSR transmission by females is substantially lower than univalent chromosome segregation in *D. melanogaster*, which is mendelian [26]. This reduced transmission is caused by the loss of PSR from the egg’s nuclear material as it enters meiosis. The magnitude of this loss measured microscopically roughly matches the proportion of adult F1 progeny that did not receive a copy of PSR. We emphasize that PSR’s loss during meiosis stands in contrast to the previously observed behavior of univalent chromosomes in *D. melanogaster*, which remain within meiotic nuclei and segregate properly to progeny [26]. Thus, our work reveals strong PSR drag in the female wasp, which we interpret is caused by an incompatibility of the B chromosome with some aspect of female meiosis in this organism.

What might underlie this incompatibility? In *D. melanogaster*, there exists a secondary mode of chromosomal segregation known as the distributive system, which is responsible for ensuring proper segregation of homologous chromosomes that do not undergo recombination and, thus, fail to form chiasmata that help stabilize the homologs at the metaphase plate during meiosis I [33]. In the fruit fly, the distributive system is especially important for meiotic segregation of the fourth chromosome, which is essential for normal development but is mostly heterochromatic and does not undergo recombination [34]. Additionally, the X chromosome often relies on the distributive system for meiotic segregation since its single, large arm occasionally fails to establish a crossover between homologs, an error that rarely occurs for chromosomes 2 and 3, which each have two large arms. Considering our findings, it is possible that *N. vitripennis* females do not have a distributive system that would otherwise ensure the nuclear retention and segregation of PSR. Interestingly, loss-of-function mutations in the *D. melanogaster* gene *nod*, a kinesin-like protein that is essential for the distributive system, results in a similar loss of the fourth chromosome from meiotic nuclei [35]. In principle, *N. vitripennis* may be able to do without a distributive system because there are no primarily heterochromatic chromosomes in the wasp’s essential genome.

Another possibility is that PSR loss may result from the observed absence of a spindle in mature, pre-activated *N. vitripennis* eggs. In these cells, the nuclear material appears condensed, indicating entry into meiosis I, but no microtubules are visible during this time. This pattern stands in contrast to the mature eggs of *D. melanogaster*, which are arrested during metaphase of meiosis I and have a well-formed spindle. The spindle-less *N. vitripennis* eggs are reminiscent of primordial oocytes in mammals, which are formed before birth and held in a prophase I state until puberty, when meiosis resumes to meiosis II upon ovulation and finishes upon fertilization [36]. In *N. vitripennis*, well-formed spindles were observed in freshly laid eggs, indicating that activation, triggered by egg laying, causes spindle formation and progression through the remainder of meiosis. Without the presence of spindle fibers in mature, pre-activated wasp eggs, PSR may lack the ability to remain associated with the nuclear mass. The essential chromosomes may be stabilized within the nuclear mass at this time due to homolog pairing and crossing over.

The second aspect of B inheritance examined here is whether PSR’s copy number can increase above one. The answer appears to be no. From crosses between PSR+ parents, the proportion of F1 progeny carrying two PSR copies at the early embryonic stage was remarkably close to the expected proportion. However, there were no adult F1 progeny from these same parents that contain more than one copy. We observed a heightened portion of progeny produced by PSR+ parents that fail to transition from the larval stage to the pupal stage. This observation is consistent with the possibility that the presence of two PSR copies causes developmental arrest at this time. A previous study showed that PSR expresses ~70 genes in the testis, one of them, *haploidizer*, playing an essential yet mysterious role in PGE [23]. It is possible that *N. vitripennis* is dosage sensitive to the expression of *haploidizer* or perhaps other PSR-expressed genes, which may reach toxic levels in somatic cells when expressed at more than their normal levels from two B copies. Such dosage sensitivity is reminiscent of sex-linked genes in other organisms like *D. melanogaster*; these genes must undergo dosage compensation to mitigate this effect [37,38]. In this scenario, wasp embryos with two PSR copies would be viable because they have not yet undergone zygotic genome activation, which occurs in most organisms during or just after the mitotic cleavage divisions [39]. Our experiments do not let us rule out the possibility that, alternatively, one of the two PSR copies is lost or dispelled from each nucleus sometime between early embryogenesis and formation of the adult germ line. However, given the unlikelihood of this possibility, the fact that it would not explain the problems we observed in larval development, and the precedence of gene dosage sensitivity, the lethality model seems much more plausible.

Together, these findings provide a more complete understanding of PSR transmission. The most visible element of PSR’s inheritance is its PGE activity, which converts female-destined (*i.e*., fertilized) eggs into PSR-carrying males. PGE is a potent mechanism for B drive given the high rate of fertilization in *N. vitripennis*. The meiotic drag effect revealed here is normally masked by the strong female-to-male converting effect; as a result, PSR is rarely present in females, and thus not normally transmitted by them. However, PSR+ females are expected to arise naturally from spontaneous loss-of-function mutations in *haploidizer*. The meiotic drag effect would hinder the transmission of such non-driving PSR variants, thus helping to maintain strongly driving Bs in the population. Meiotic drag would also provide fitness benefits to the wasp by reducing the frequency of doomed progeny having two PSR copies. Broadly, our work reveals unforeseen properties of PSR and the organism’s reproductive biology that help govern the successful inheritance of this extreme selfish genetic element.

## Materials and methods

### Perpetuation of the PSR line

The PSR chromosome was kept in the wild-type wasp line, *AsymC*, which is derived from the *Labii* strain that was antibiotically cured of bacterial symbionts [40]. In each generation, PSR-carrying *AsymC* males were crossed pairwise with virgin *AsymC* females and set on *Sarcophaga bullata* hosts (Carolina Biological Supply, catalog # 173486) for 2–3 days at 25°C. After F1 adult emergence, all-male broods were kept for further propagation while female-biased broods were tossed.

### The production and genotyping of PSR-carrying females

PSR+ females were generated using a procedure that has been previously described in detail [23]. In essence, an ~800 bp region of the full-length *haploidizer* cDNA was amplified with primers containing the T7 viral promoter sequence. The resulting PCR product was column-purified using the Qiaquick PCR purification kit (Qiagen, catalog # 28104) and used as a template to perform bidirectional transcription with the Invitrogen MEGAscript RNAi kit (ThermoFisher Scientific, catalog # AM1626). Following clean-up, the dsRNA was microinjected into *AsymC* PSR+ male pupae in the yellow body and red-eyed developmental stage. Once these sRNAi-treated males enclosed as adults, they were crossed pairwise with *AsymC* females. Individual broods were screened for the appearance of F1 females. To confirm that the females carry PSR, genomic DNA was extracted from a few of these individuals in each female-containing brood using the DNeasy Blood and Tissue kit (Qiagen, catalog# 69504). PCR using primers specific for *haploidizer* were used under standard amplification conditions and 50-fold diluted gDNA as a template. The sequences of these primers are: (4317-Forward) 5′-GCG ACA GCC ACC GAA TTT AC-3′ and (4317-Reverse) 5′-GAC GTG CAA AAA CCT GCA TCT-3′.

### Fitness testing of PSR+ females

The fitness of *AsymC* PSR+ females was measured by assessing four characteristics—body size, longevity, F1 egg hatch rate, and F1 adult progeny produced per female—in comparison to age-controlled, wild-type *AsymC* females. All four characteristics were measured from the same batch of PSR+ or wild-type females to minimize variation among broods. Upon eclosion, females were fed a mixture of 50% honey in water and allowed to feed on fresh *Sarcophaga bullata* pupae for 24 hours. Females were temporarily immobilized with CO_2_, and digital images were taken of each female at the same magnification using a dissecting microscope. The length from the tip of head to the tip of abdomen was measured using Adobe Photoshop. The females were then individually placed into separate vials, and each was given a couple of fresh *S. bullata* pupae. They were allowed to oviposit into the hosts for 48 hours and switched to new hosts twice in successive 48-hour periods. Host pupal shells were opened immediately following female removal and the number of eggs was counted. They were then incubated at 25°C for 30 hours in a humidity chamber to score the number of eggs that hatched into larvae. The progeny were then incubated at 25°C until they emerged as adults (over two weeks). The number and developmental stage of F1 progeny were scored for each pupa. For longevity assessment, the same females were kept on hosts (changed to fresh hosts every 3 days) at 25°C and monitored every day until life expiration.

### Developmental profiling of PSR+ progeny

To monitor the developmental progression of *N. vitripennis* progeny, PSR+ males were mated with either WT females or PSR+ females. Following mating, the females were individually placed onto hosts and allowed to oviposit for 4 hours. The females were removed and allowed to oviposit into fresh hosts two more times. Immediately after each oviposition period, a portion of the host’s shell was removed, and the wasp eggs were carefully counted. The hosts were then placed into a humidity chamber and allowed to develop for 30 hours at 25°C. After this time, each host was scored for the number eggs that hatched into first instar larvae. Following nine additional days in the incubator under humidity, the hosts were opened completely and carefully to avoid perforating the remaining host carcass so all progeny could be scored; by this time, all progeny still living will be third instar larvae or pupae. The progeny were saved and monitored for another week, giving them ample chance to complete development.

### Wasp embryo collection and fixation

Mature wasp eggs were collected by dissecting whole ovaries from gravid females in 1×PBT buffer. The mature eggs were removed from the rest of the ovary tissue with ultrafine forceps and chemically preserved for exactly 30 min in a mixture of 3 mL heptane, 1.5 mL 1×PBS, and 600 µL 37% paraformaldehyde. The fixative was removed, and the preserved eggs were washed twice with 1×PBT to remove any traces of the fix solution. The eggs were then placed in a droplet of 1×PBT on the surface of a plastic Petri dish lid and lanced in half with a 30-gauge hypodermic needle (this step is necessary for allowing penetration of the staining reagents described below). To collect young embryos, gravid female wasps were allowed to oviposit into the anterior end of host pupae for 1.5–2 hours. Embryos were carefully collected from the hosts using an egg pick (a single side of a pair of forceps) and fixed in the same fixative solution as used for eggs. The embryos were removed from the fixative with a pipette tip and transferred onto a small piece of Whatman paper, blotted dry, and adhered to double-sided clear tape in the bottom of a small plastic Petri dish. The eggs were hydrated with ~1 mL of 1×PBT and rolled out of their vitelline envelopes using a 30-gauge needle. The devitellinized embryos were then transferred into a microfuge tube for staining.

### Antibody staining, fluorescence in situ hybridization, and confocal microscopy of whole-mount tissues

A mouse anti-αTubulin antibody (Santa Cruz Biotech) was used at 1/100 to stain the spindle apparatus, and a mouse anti-Lamin Dm0 antibody (Developmental Studies Hybridoma Bank) was used at 1/50 to visualize the nuclear envelope. To perform antibody staining, fixed tissues were incubated overnight in primary antibodies at 4°C under slow rotation. After three 10 min washes in 1×PBT, tissues were counterstained on a platform rocker at room temperature with Alexa488-conjugated anti-mouse IgG secondary antibodies (ThermoFisher Scientific) for 1 hr. The tissues were then washed 3 times for 10 min in 1xPBT and mounted in Vectashield mounting medium containing 4′, 6-diamidino-2-phenylindole (DAPI) (Vector Laboratories, H-1200-10). To perform DNA FISH on tissues that have been antibody-stained, the tissues were post-fixed in 4% paraformaldehyde in 1xPBT for 30 min at room temperature and washed 2× in 2×SSCT buffer. The tissues were then treated with a series of 2×SSCT solutions containing increasing amounts of formamide, followed by an overnight incubation in 1.1× hybridization buffer with diluted probe. Subsequently, tissues were taken through the same series of 2×SSCT solutions with formamide in decreasing amounts, washed twice with 2×SSCT, and mounted on a slide. The probe used to visualize PSR was synthesized and conjugated with Cy3 by IDT DNA, ; the sequence, recognizing the high-copy repeat PSR22, is: 5′-CAC TGA AAA CCA GAG CAG CAG TTG AGA-3′. This description of DNA FISH captures the major steps of a more detailed, previously published protocol [41]. Image collection was performed using a Leica SPE DMIRB inverted fluorescence confocal microscope. Most images represent collapsed Z series to capture cellular objects in different fields of view. Images were exported as JPEG or TIFF files for figure-building using Adobe Photoshop.

### Preparation and microscopic analysis of squashed sperm cells

To determine the copy number of PSR in mature sperm cells, we dissected reproductive tracts from F1 males produced from crosses between two PSR+ parents. These tissues were fixed in a 15 µL drop of 45% acetic acid and 2.5% paraformaldehyde on a cover slip for exactly 4 min and then squashed between the cover slip and a microscope slide. After freezing the slide in liquid nitrogen, the cover slip was quickly flicked off at one of its corners with a razor blade, and the slide with adhered, squashed tissue was soaked in 100% ethanol for 10 min, and air dried. For DNA FISH, the PSR probe (see above) was diluted to 100 ng/µL in 1.1×hybridization buffer and placed onto the tissue spot on the slide, and a cover slip was added. The slide was then heated on a hot block for 5 min at 95°C and then incubated overnight at 30°C in a humidity chamber. Subsequently, the cover slip was removed, and the slide was washed 3 times in 2×SSCT before mounting in Vectashield. Imaging of the mature sperm cell nuclei was performed on a Leica DM4000 B epifluorescence microscope. Collected images were exported and processed as described above.

## Supporting information

S1 Data Raw Images. Original agarose gels showing PCR amplification of a PSR-specific locus, PSR4317 (top gel) and a control gene, rp49 (bottom gel), across several individuals. The lane labels are shown in Fig 1A.
(PDF)

S2 Data Original data collected for body size, longevity, egg production, and number of offspring produced per female. (XLSX)

S3 Data Original agarose gels showing PCR genotyping for PSR status in individual F1 progeny laid by unmated PSR+ females (top five gels) and by PSR+ females mated with wild-type males (bottom five gels). (PDF)

S4 Data Original female sex ratio measurements used to generate graphs shown in Fig 2B. (XLSX)

S5 Data Depiction of PSR segregation during meiosis and the first embryonic mitotic division. PSR placement on the metaphase spindles of the first and second divisions are shown. PSR is expected to segregate equally to either the left or right side of the spindle during the first division. In the top scenario, PSR will end up in the egg’s nucleus and, thus, will be inherited. In the bottom scenario, PSR will reside only in the polar bodies and will not be inherited. Each scenario is equally likely so long as PSR undergoes normal segregation.
(TIF)

S6 Data Original microscopic images of 0–2-hour embryos laid by unmated PSR+ females to assess why maternal transmission to progeny is low. PSR is red and DNA is gray. Both channels are merged.
(PDF)

S7 Data Original microscopic images of 0–2-hour embryos laid by PSR+ females mated with PSR+ males to measure PSR copy number in progeny at this early developmental stage. PSR is red and DNA is gray. Both channels are merged.
(PDF)

S8 Data Original data of scorings for PSR copy number per sperm nucleus per testis of F1 males produced by PSR × PSR parents. (XLSX)

S9 Data 0–2-hour embryos produced by F1 fathers obtained from PSR × PSR crosses. The number of PSR foci per embryonic nucleus were recorded.
(XLSX)

S10 Data Developmental profiling of F1 progeny produced by PSR × PSR and WT × PSR parents. After individual parents were set on hosts to produce progeny, they were genotyped to confirm PSR+ status. Details of profiling are found in Materials and methods.
(XLSX)

## Abbreviations

FISH: fluorescence in situ hybridization
PGE: paternal genome elimination
PSR: Paternal Sex Ratio
sRNAi: systemic RNAi
