# Supplementary material for: A male-transmitted B chromosome undergoes strong meiotic drag in females of the jewel wasp Nasonia vitripennis
Source: PLoS Biol. 2026 Jan 16;24(1):e3003599. doi: 10.1371/journal.pbio.3003599 (PMC12826520; doi:10.1371/journal.pbio.3003599)

S1 Data

Original gel for Figure 1A: Amplification with primers specific for PSR4317

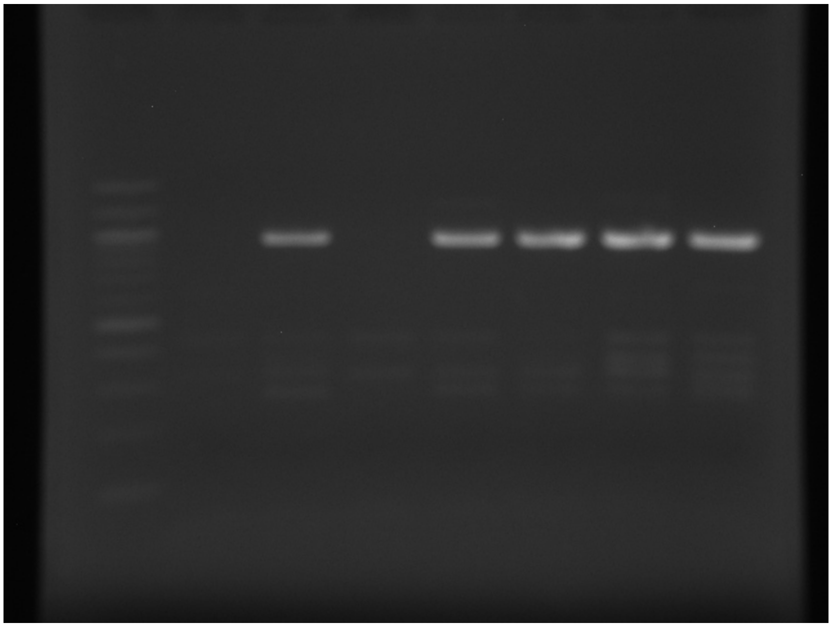

Original gel for Figure 1A: Amplification with primers specific for rp49

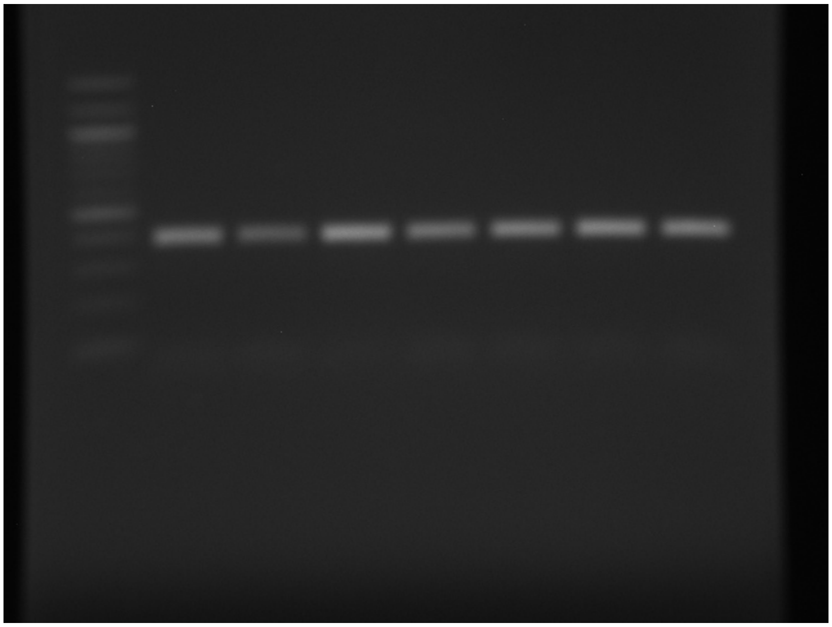

Supplement: S1 Data — Original agarose gels showing PCR amplification of a PSR-specific locus, PSR4317 (top gel) and a control gene, rp49 (bottom gel), across several individuals. The lane labels are shown in Fig 1A. (PDF) [file pbio.3003599.s001.pdf]
