# Supplementary material for: A male-transmitted B chromosome undergoes strong meiotic drag in females of the jewel wasp Nasonia vitripennis
Source: PLoS Biol. 2026 Jan 16;24(1):e3003599. doi: 10.1371/journal.pbio.3003599 (PMC12826520; doi:10.1371/journal.pbio.3003599)
Supplement: S3 Data — (PDF) [file pbio.3003599.s003.pdf]

S3 Data

PCR genotyping of F1 progeny laid by unmated PSR+ females.

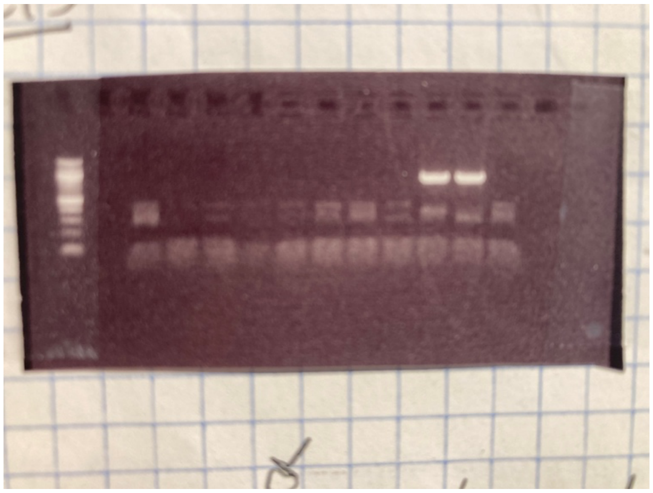

2 of 10

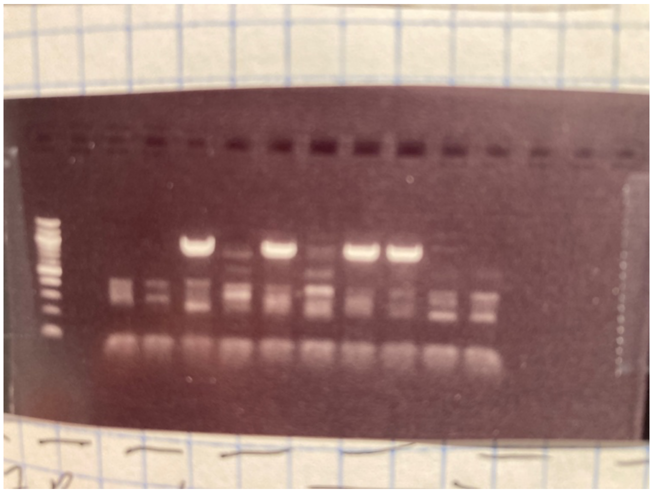

4 of 10

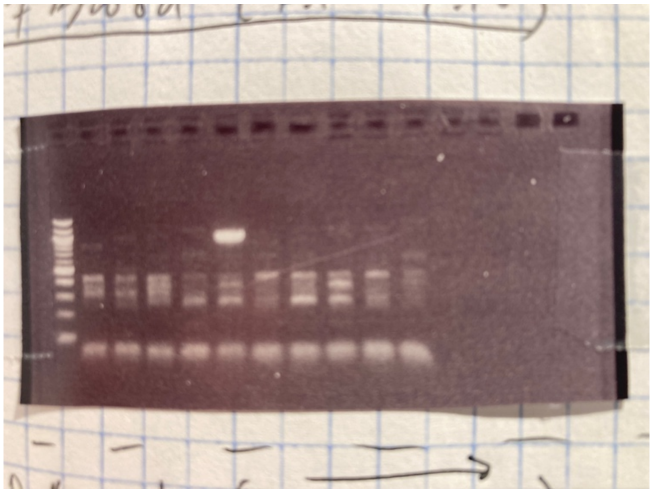

1 of 10

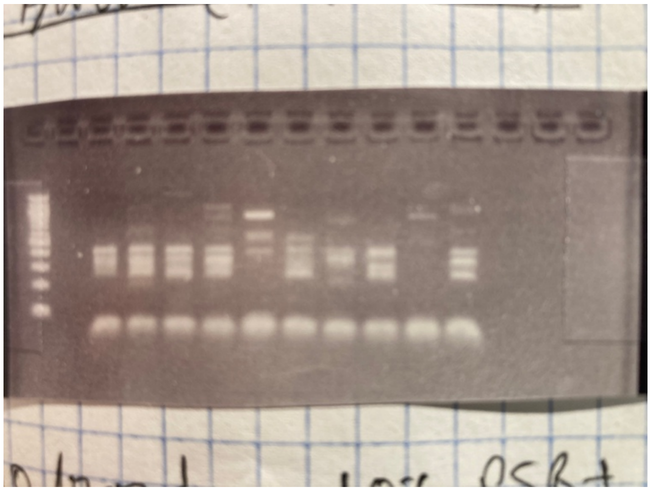

1 of 10

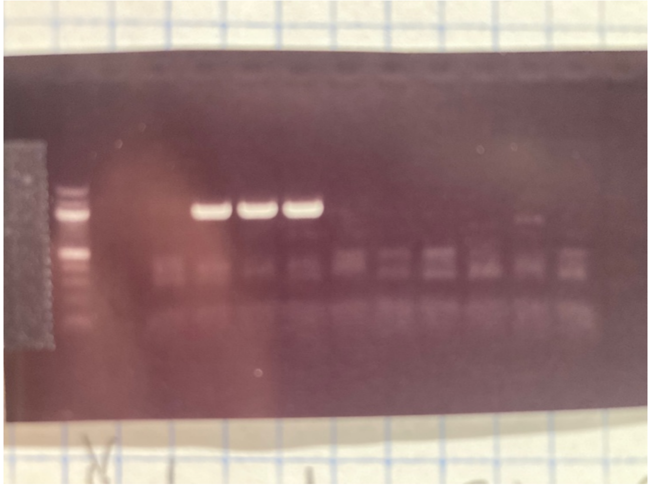

3 of 10

PCR genotyping of F1 progeny laid by PSR+ females mated with wild type males.

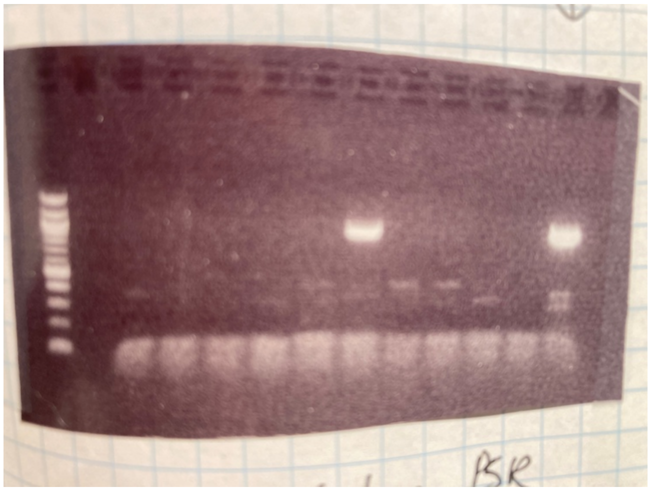
1 of 10 (last lane is a positive control)

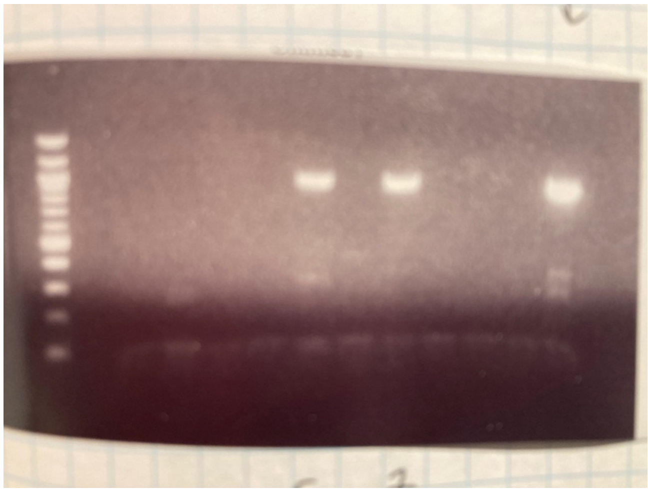
2 of 10 (last lane is a positive control)

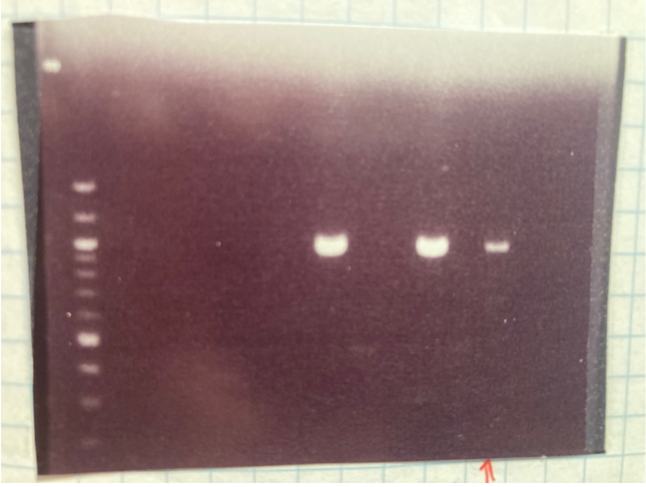
2 of 10 (last lane is a positive control)

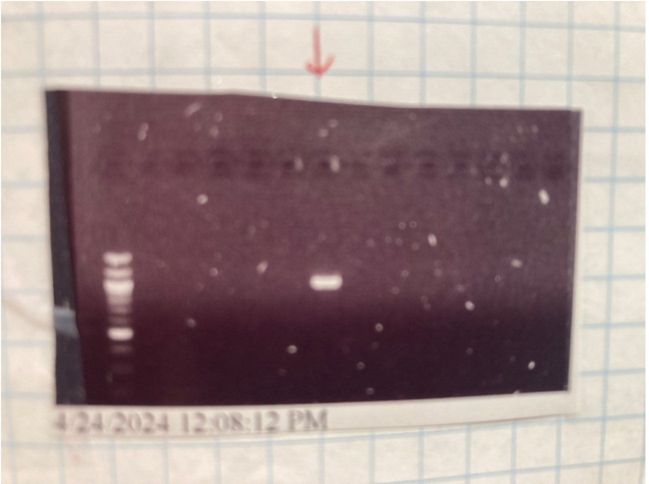

1 of 10 (no positive control on this gel)

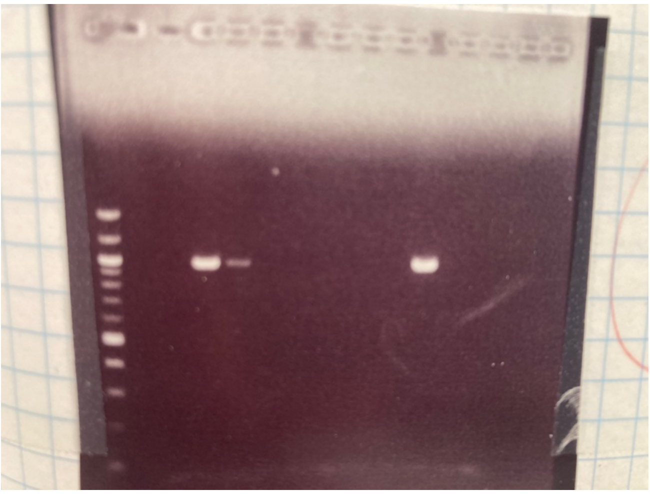

3 of 10 (no positive control on this gel)
