# Supplementary figures and images for: A male-transmitted B chromosome undergoes strong meiotic drag in females of the jewel wasp Nasonia vitripennis
Source: PLoS Biol. 2026 Jan 16;24(1):e3003599. doi: 10.1371/journal.pbio.3003599 (PMC12826520; doi:10.1371/journal.pbio.3003599)

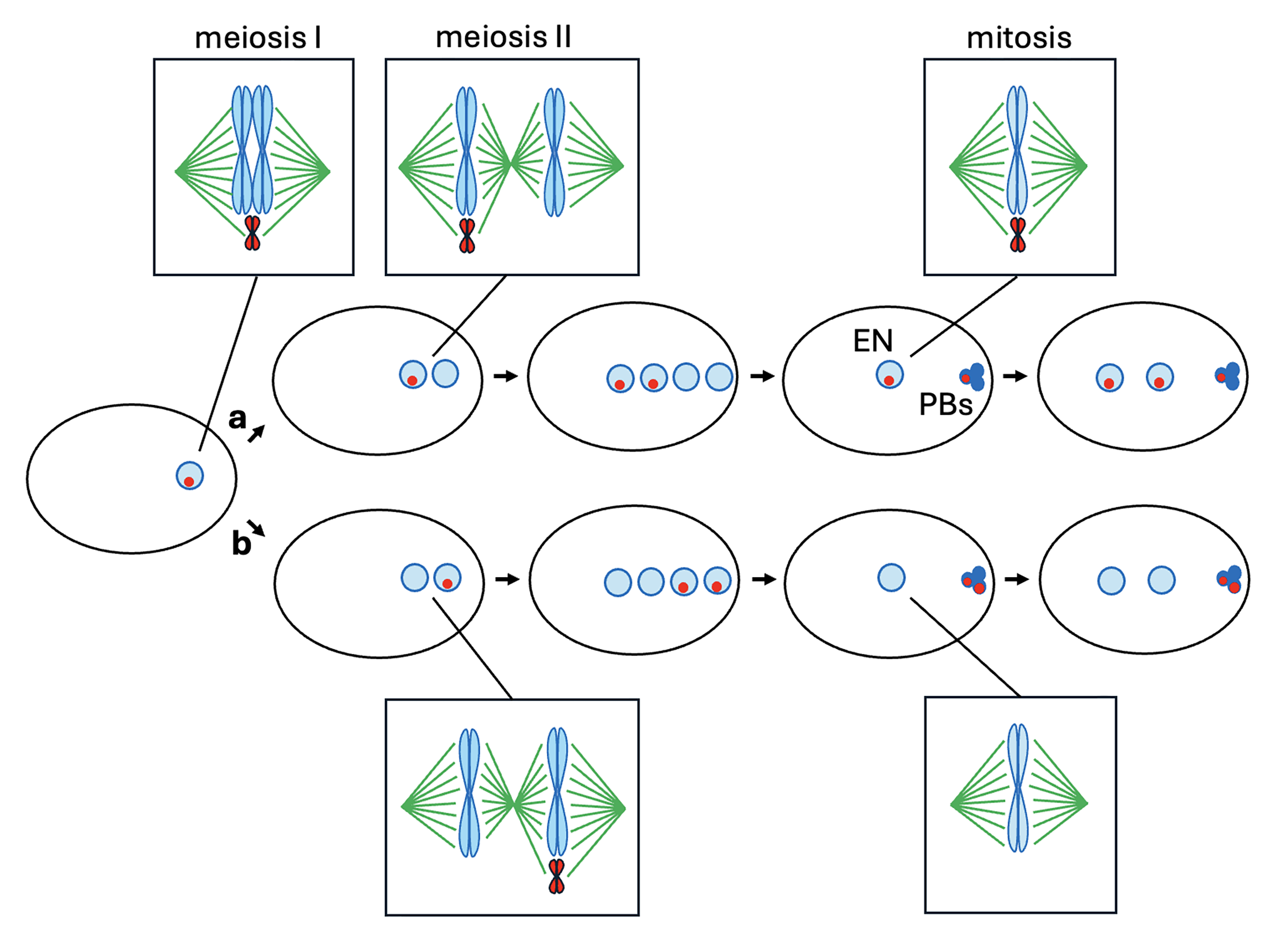

Supplement: S5 Data — PSR placement on the metaphase spindles of the first and second divisions are shown. PSR is expected to segregate equally to either the left or right side of the spindle during the first division. In the top scenario, PSR will end up in the egg’s nucleus and, thus, will be inherited. In the bottom scenario, PSR will reside only in the polar bodies and will not be inherited. Each scenario is equally likely so long as PSR undergoes normal segregation. (TIF) [file pbio.3003599.s005.tif]
