# Supplementary material for: A male-transmitted B chromosome undergoes strong meiotic drag in females of the jewel wasp Nasonia vitripennis
Source: PLoS Biol. 2026 Jan 16;24(1):e3003599. doi: 10.1371/journal.pbio.3003599 (PMC12826520; doi:10.1371/journal.pbio.3003599)

S5 Data

0-2hr embryos laid by unmated PSR+ females to investigate why maternal transmission to progeny is low. PSR is red, DNA is grey. Both channels are merged.

Images 001-011, taken 3-20-24

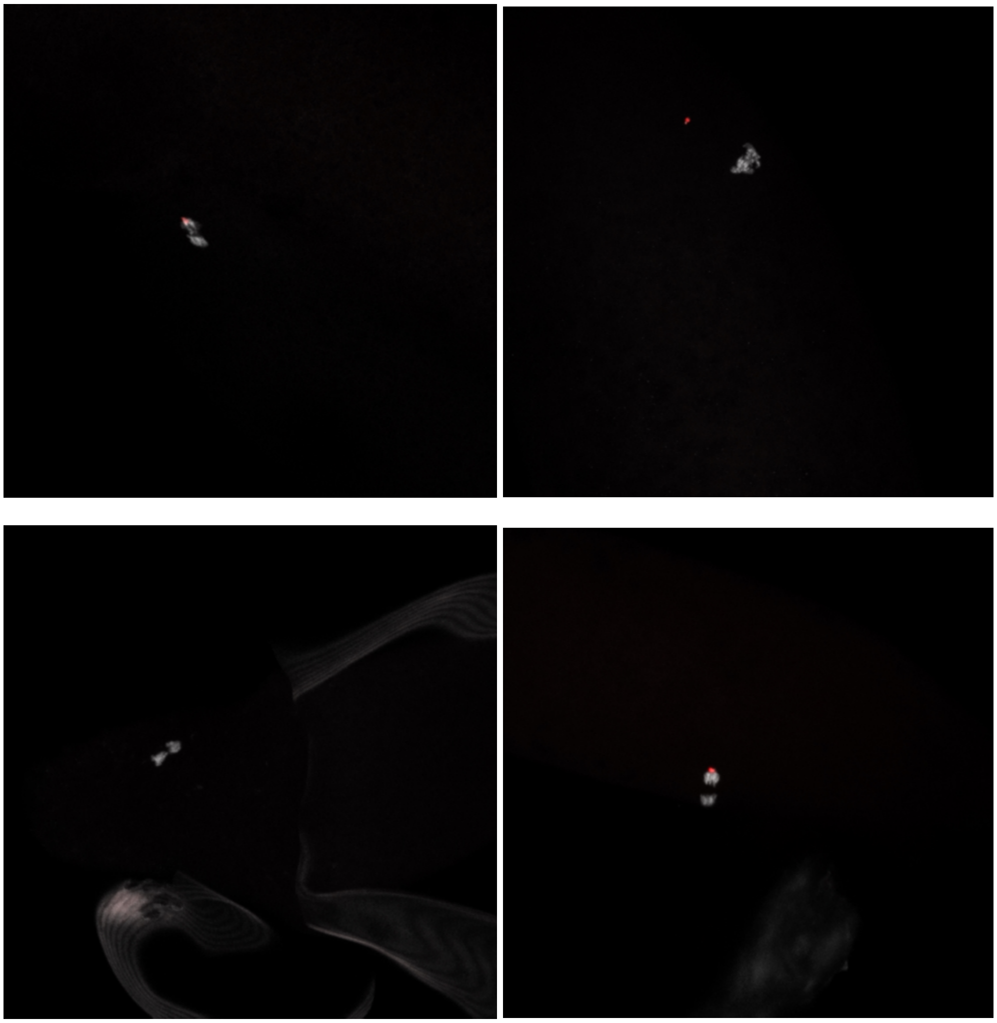

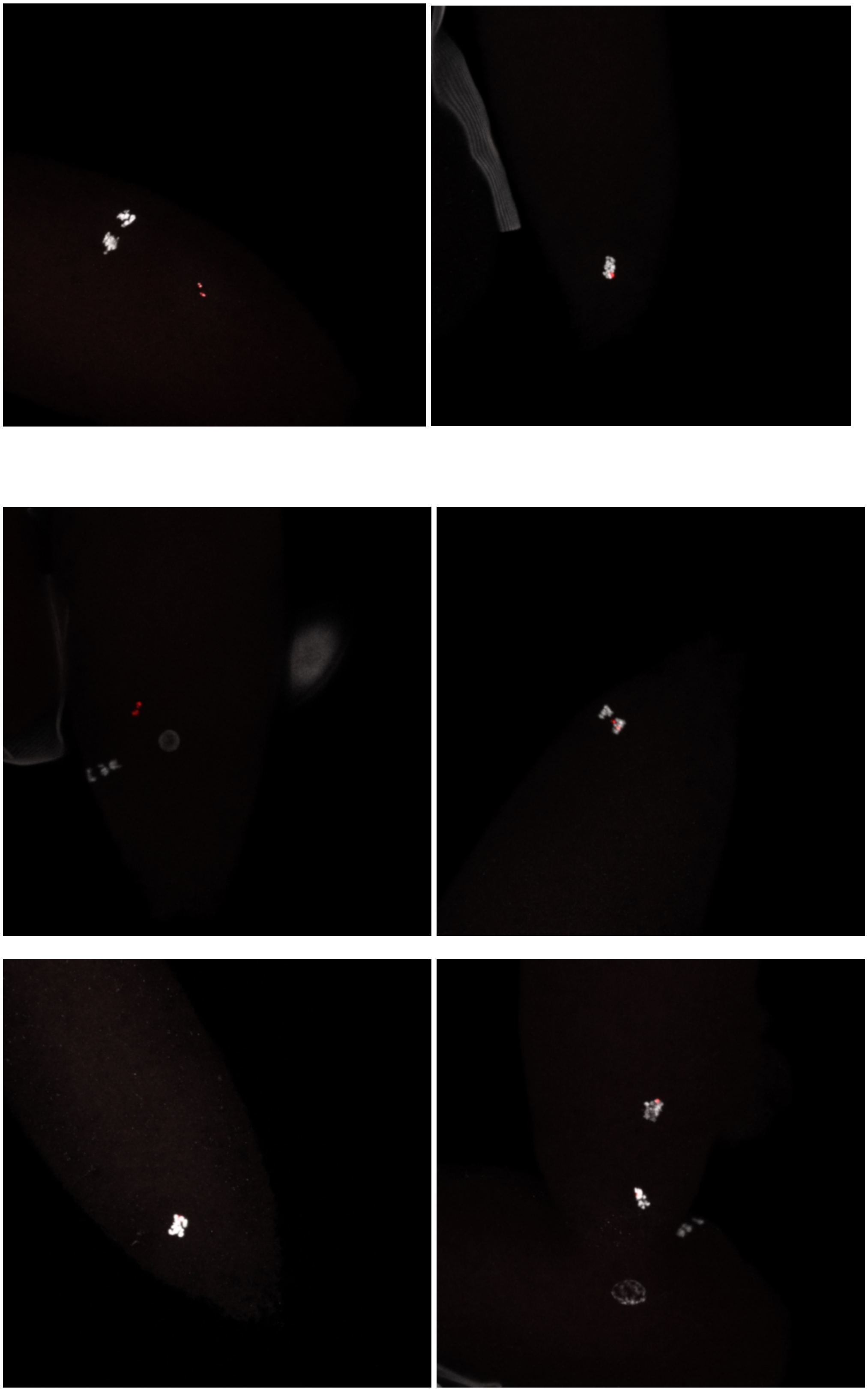

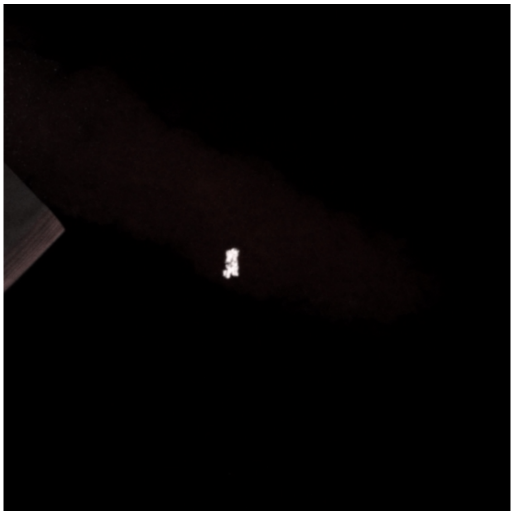

Images 001-012, taken 11-01-23 first set

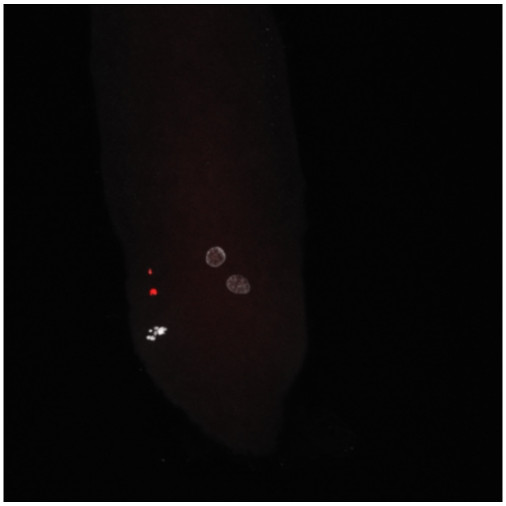
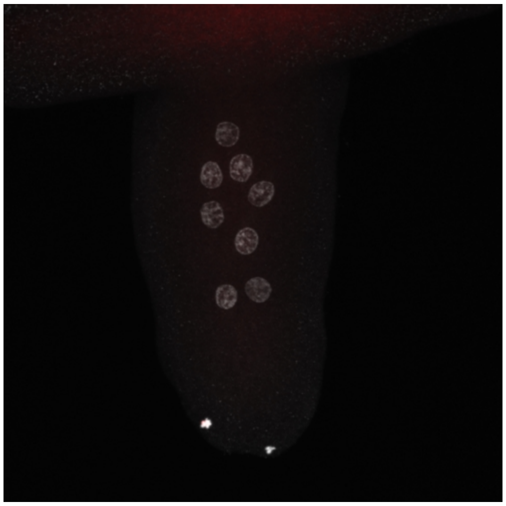
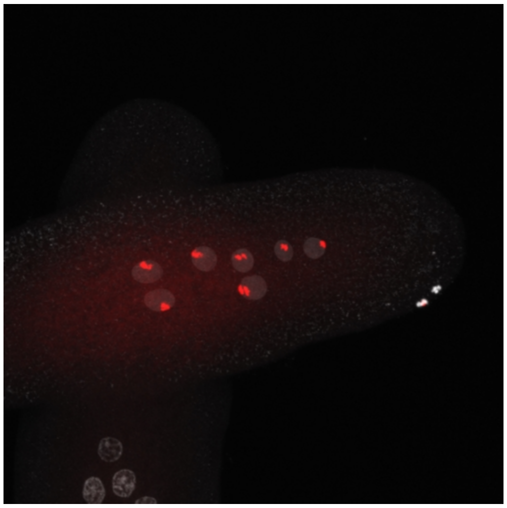
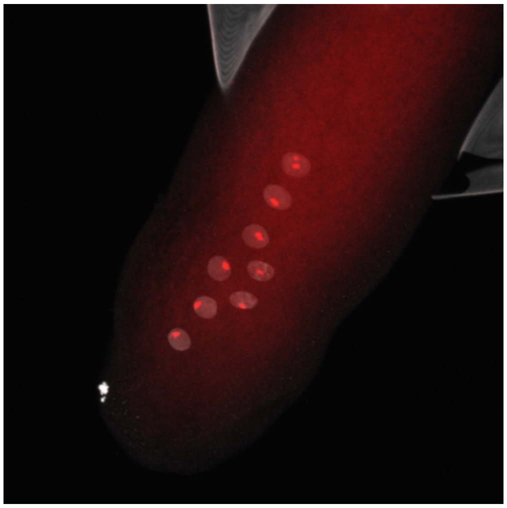

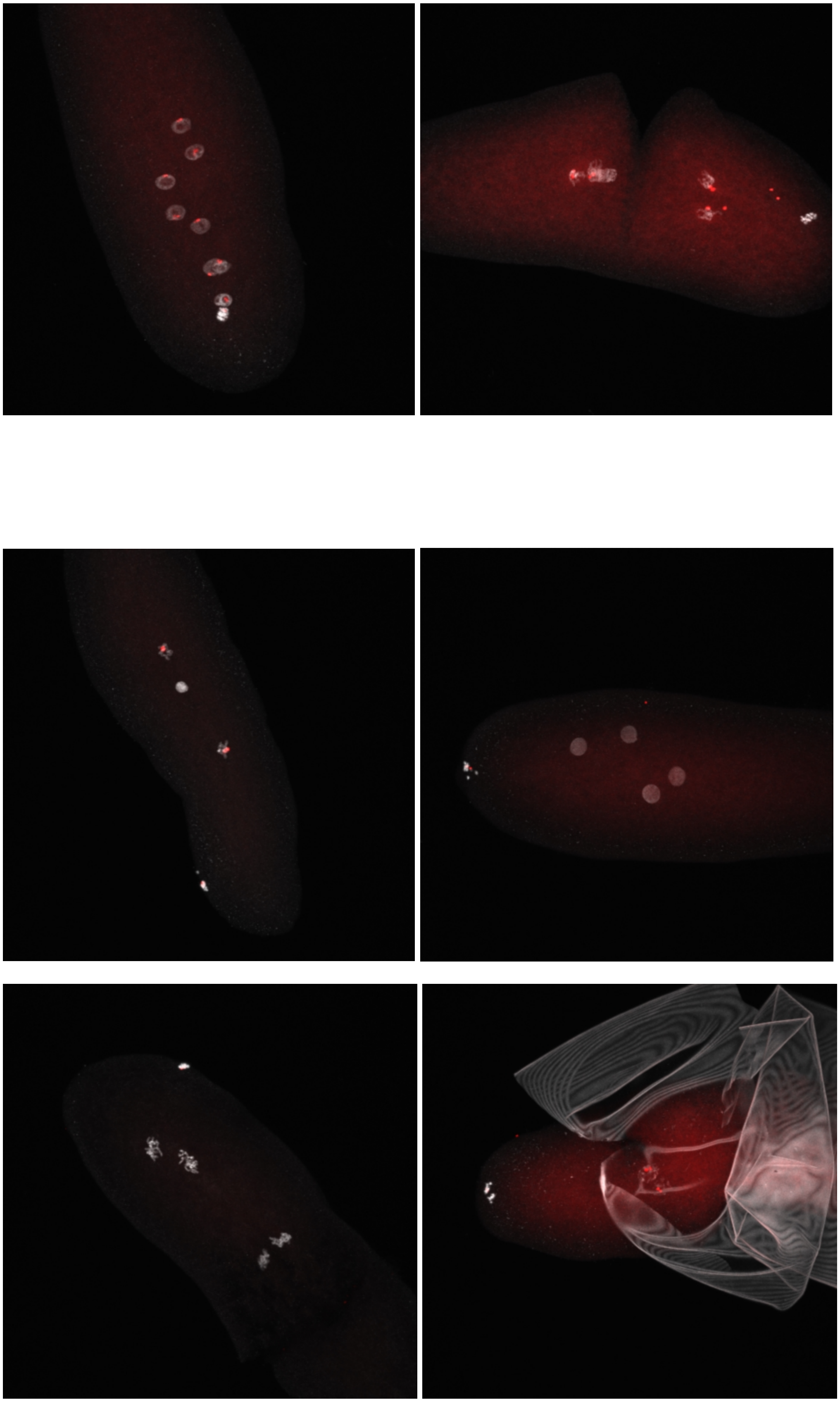

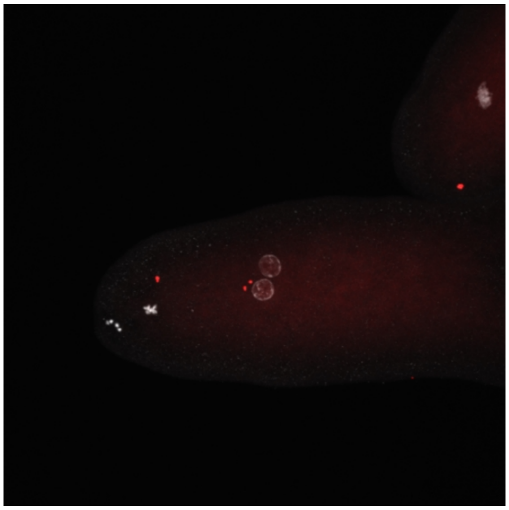
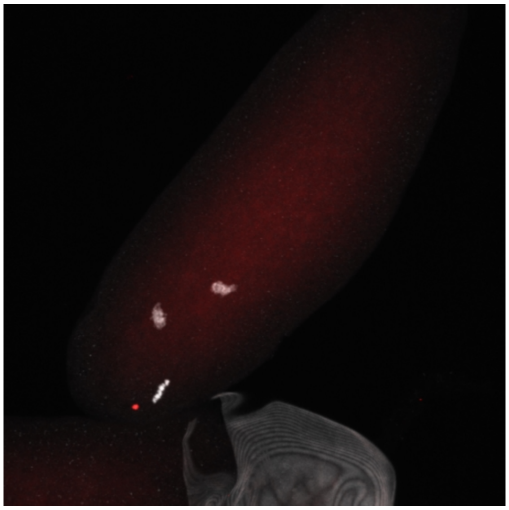

Images 001-025, taken 11-01-23 second set

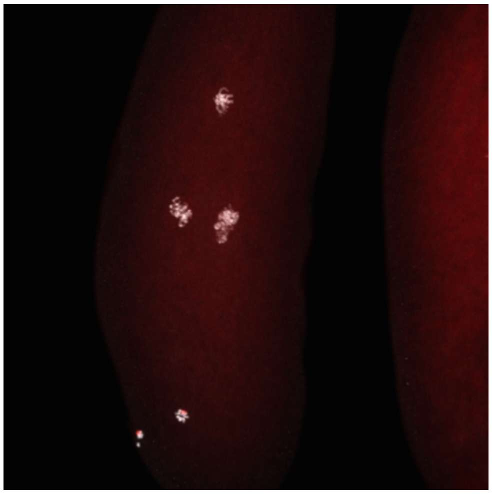
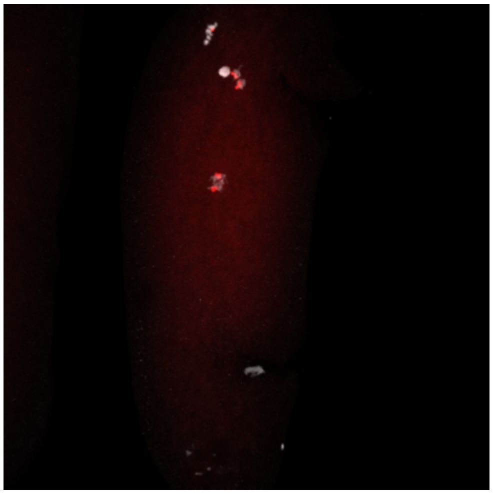
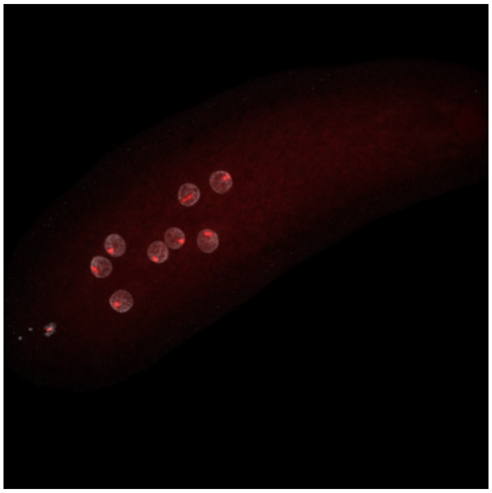
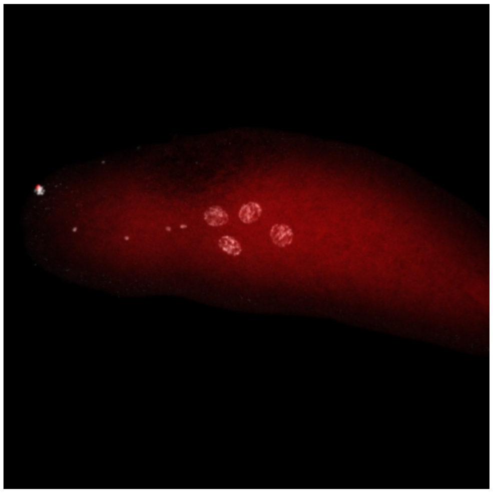

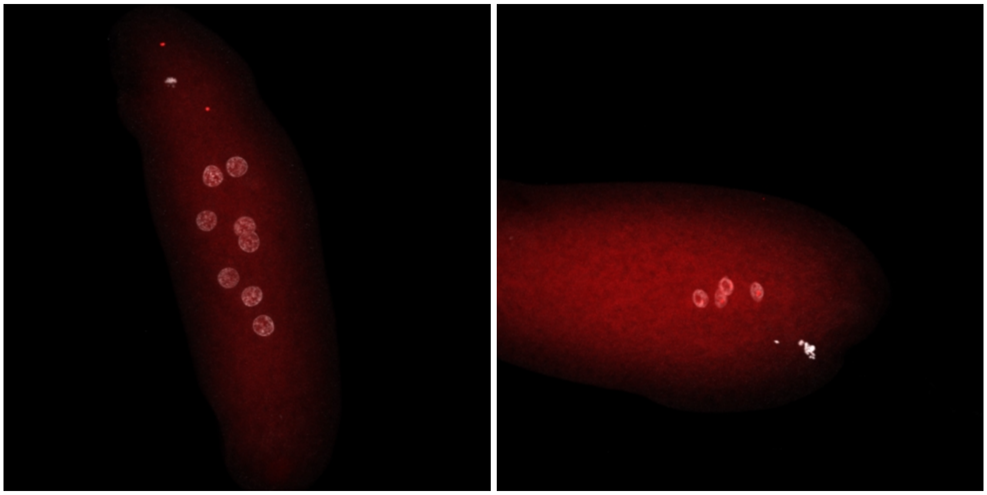

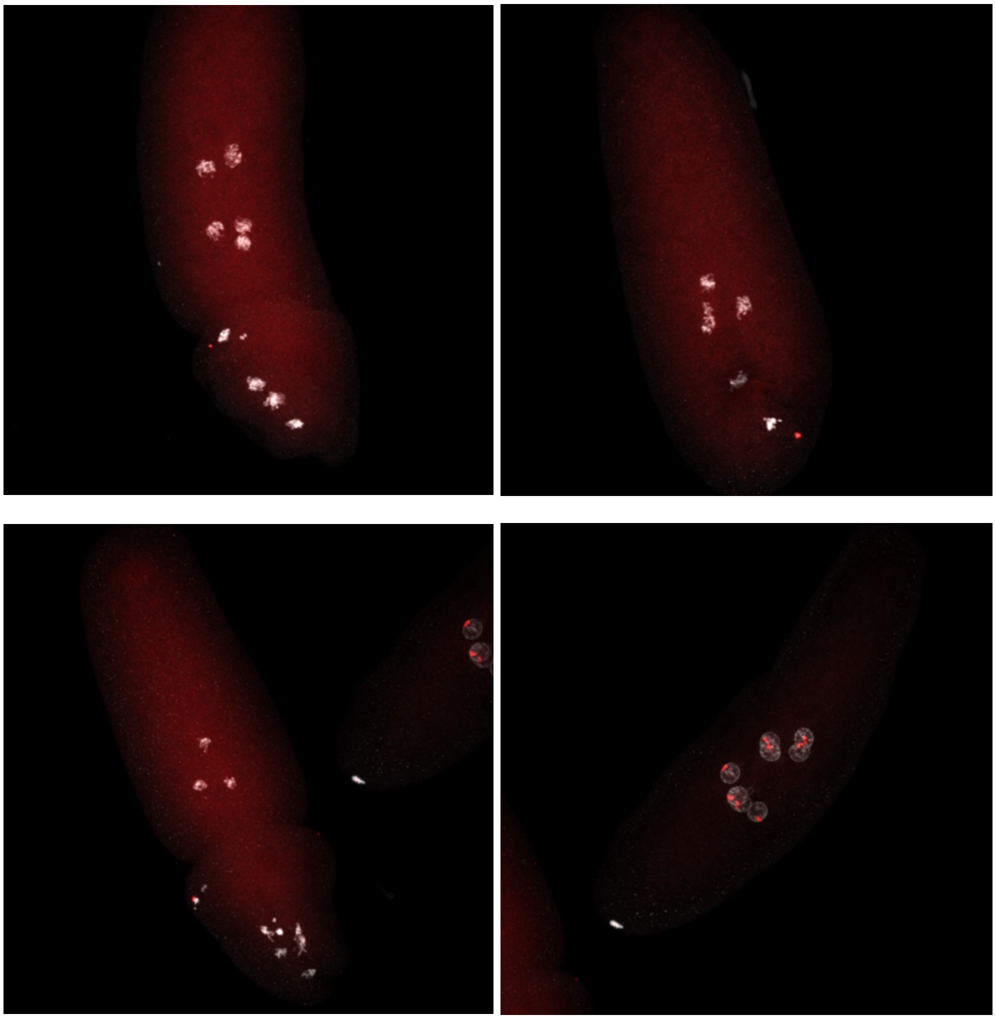

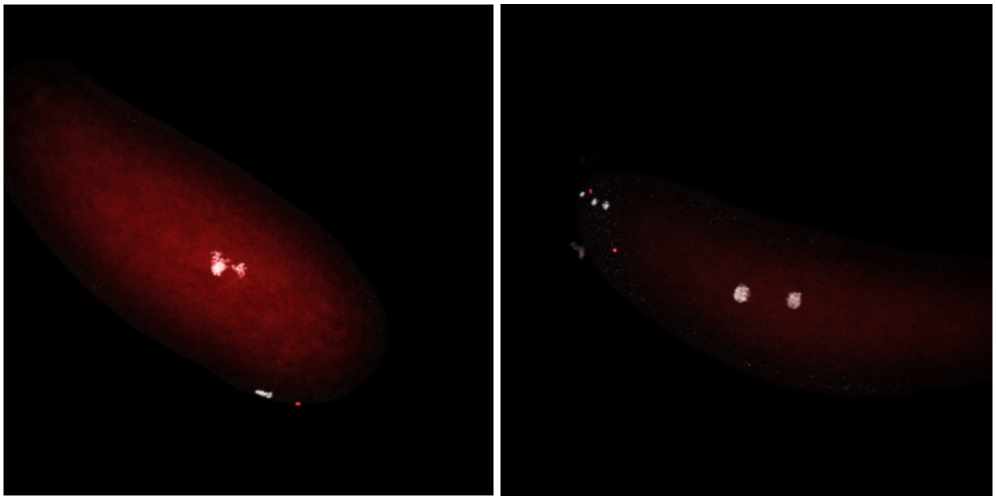

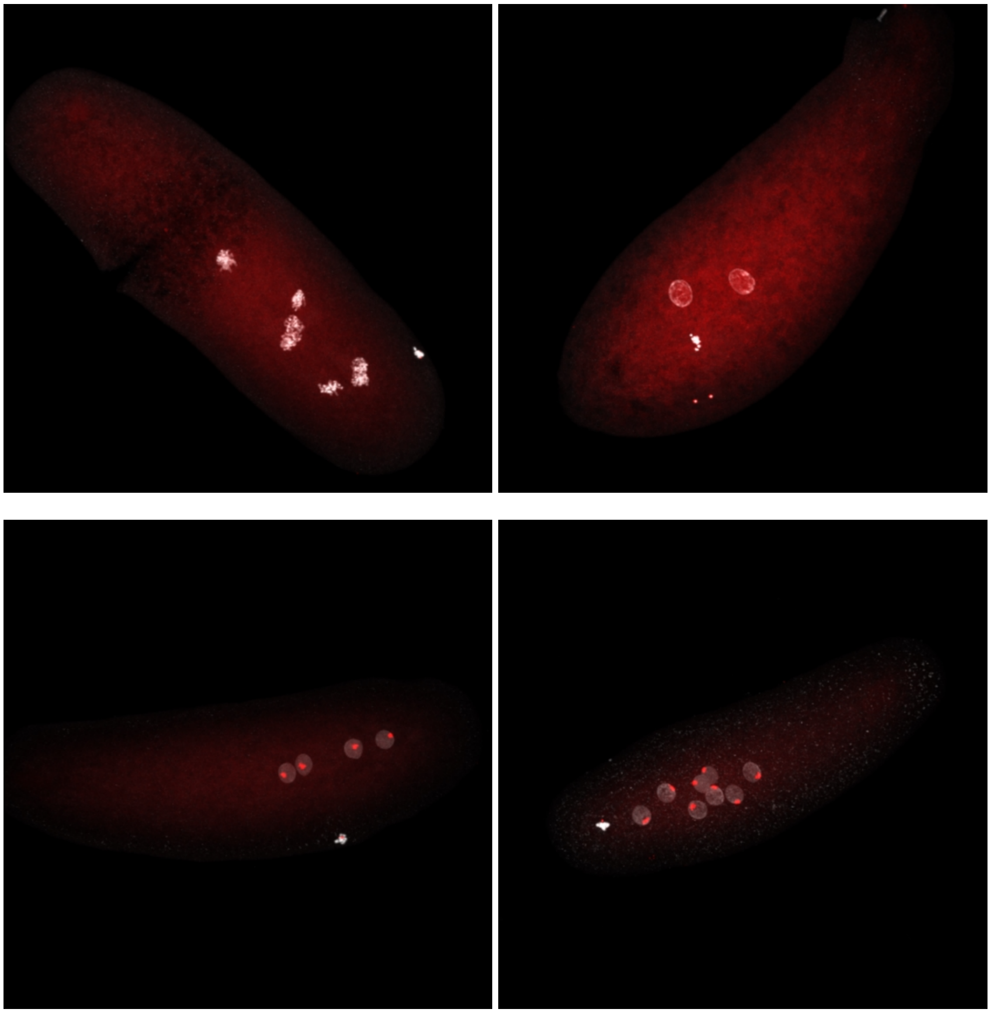

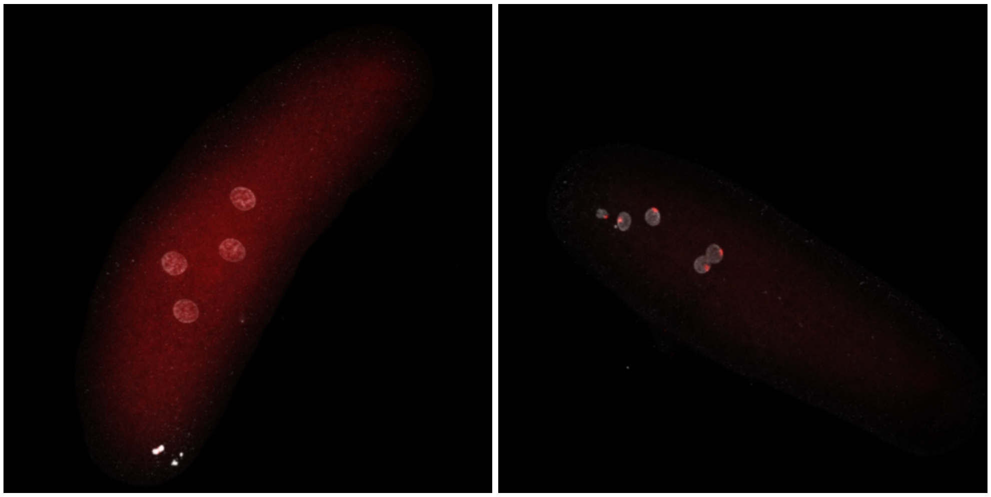

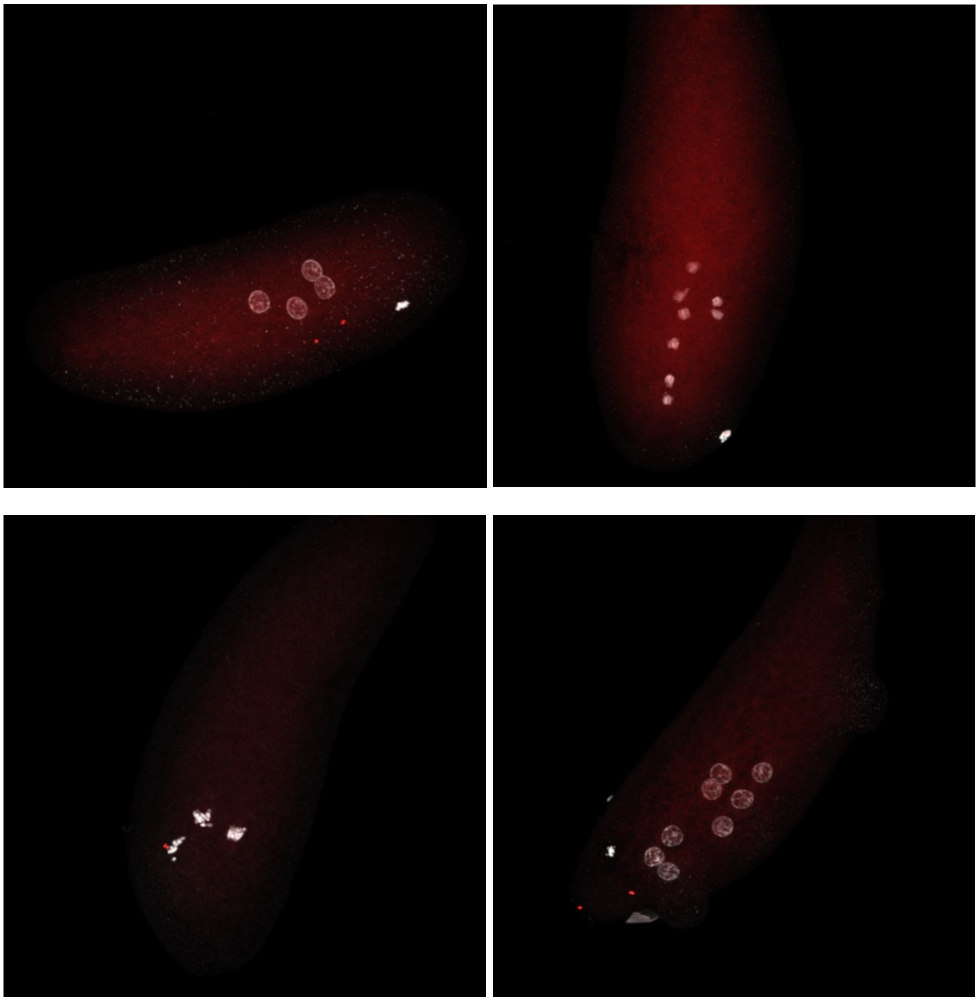

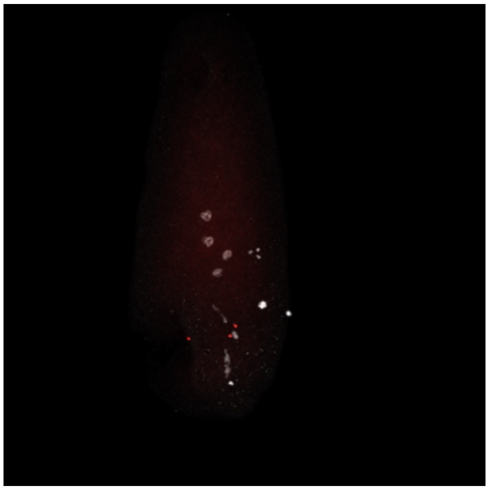
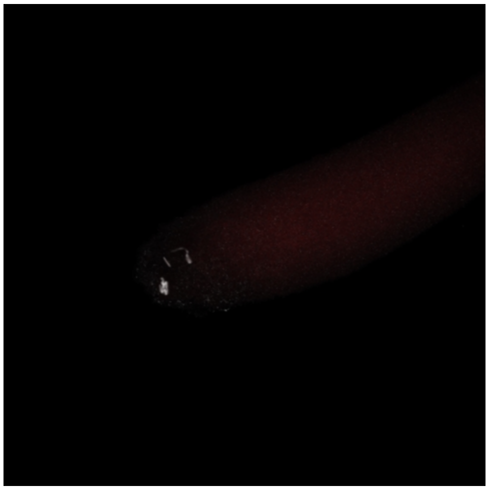

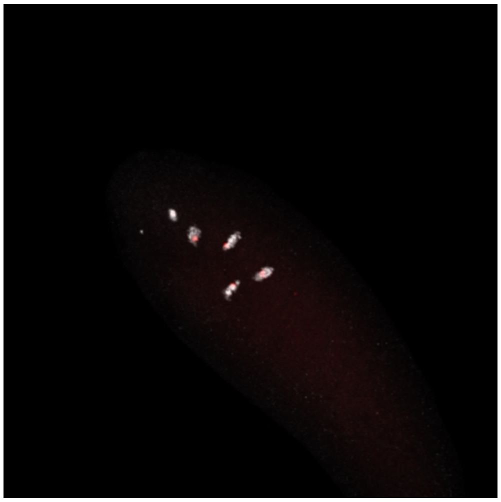

Images 001-030, taken 11-01-23 third set

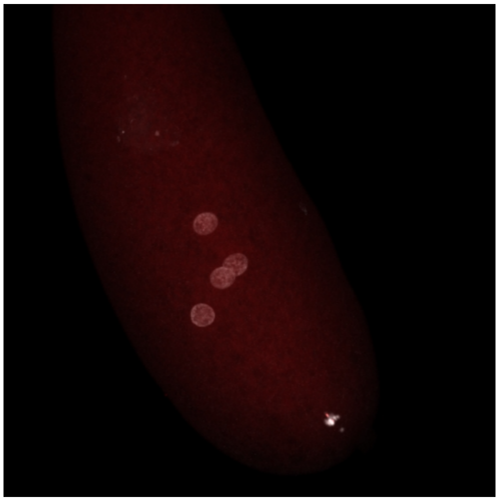
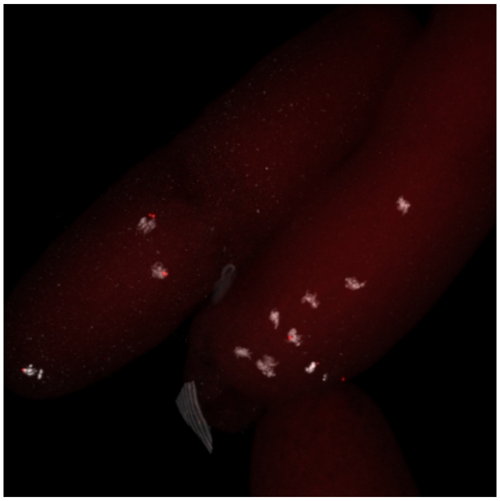

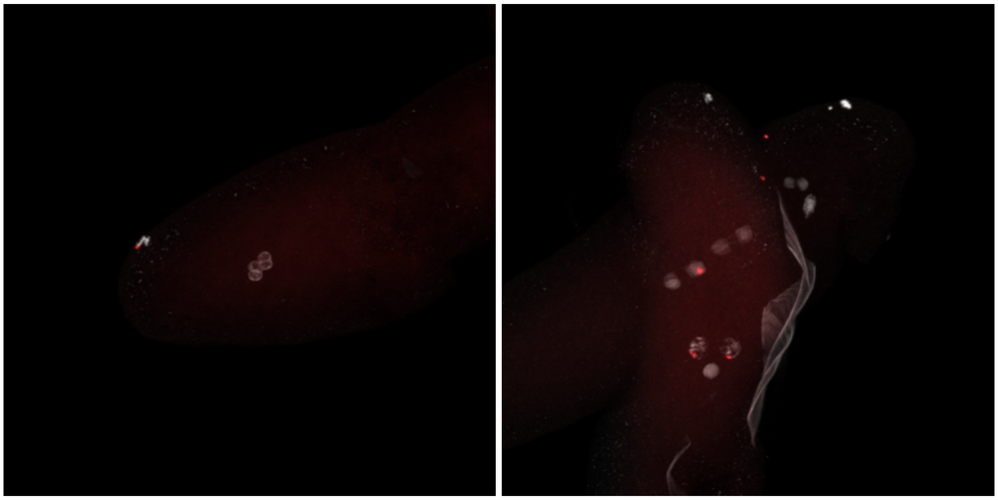

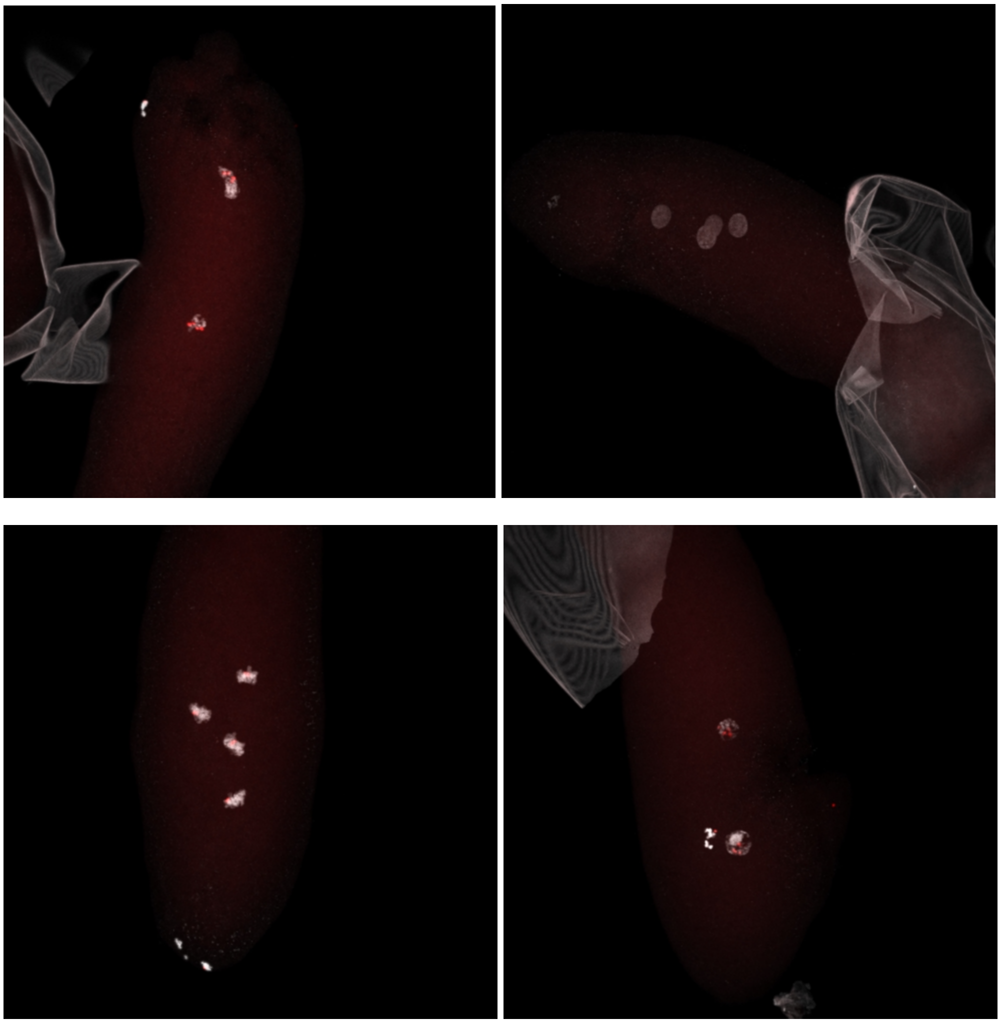

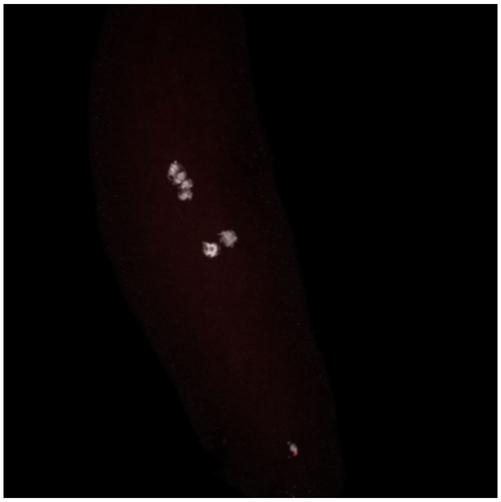
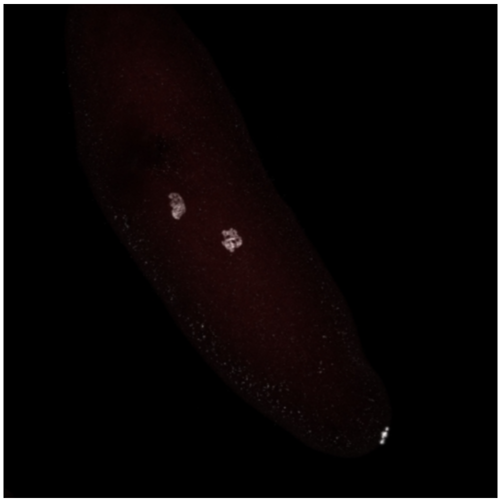

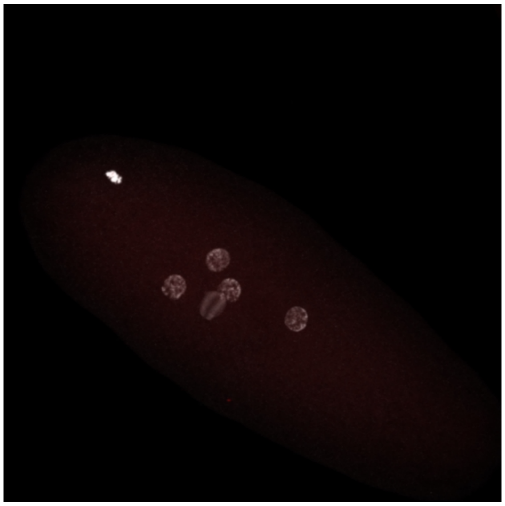

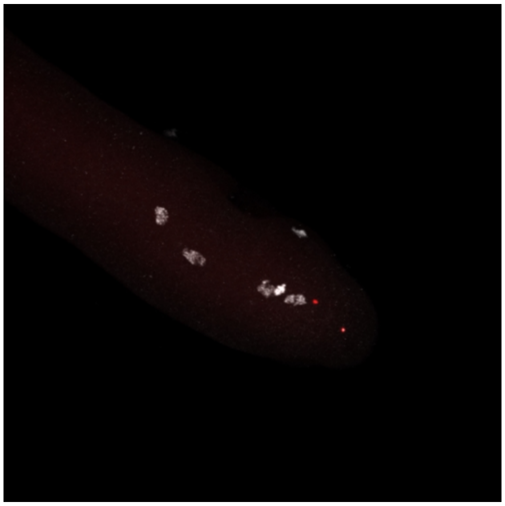
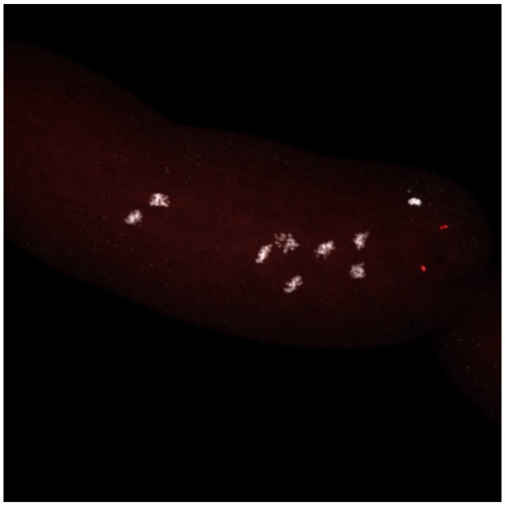

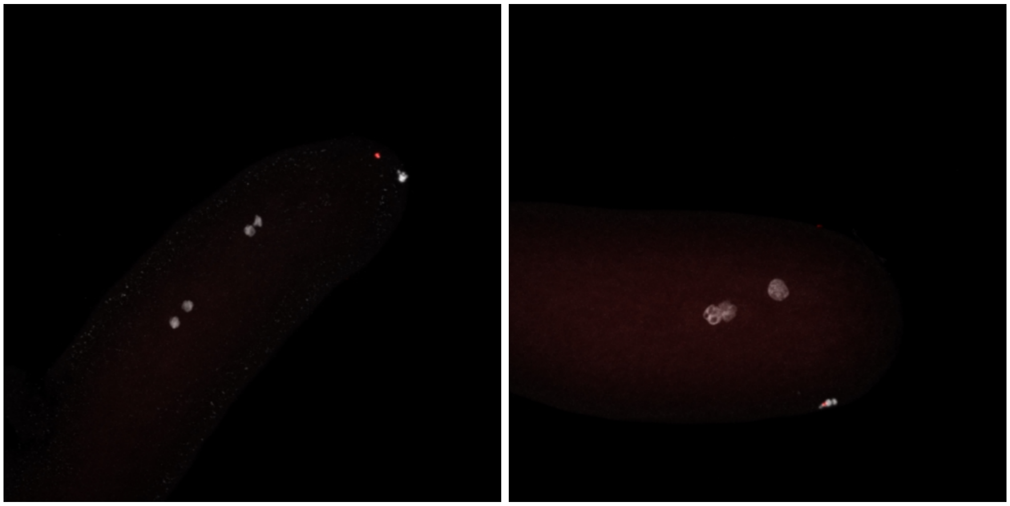

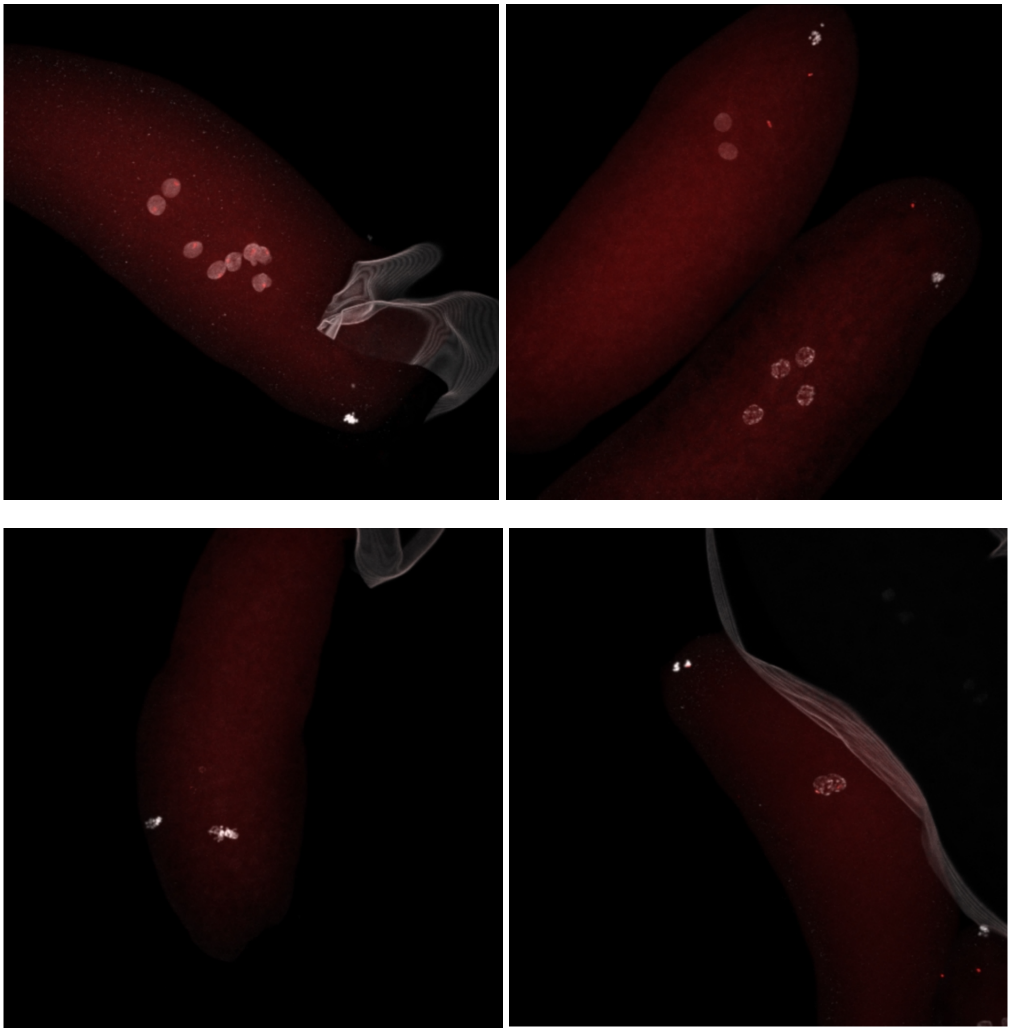

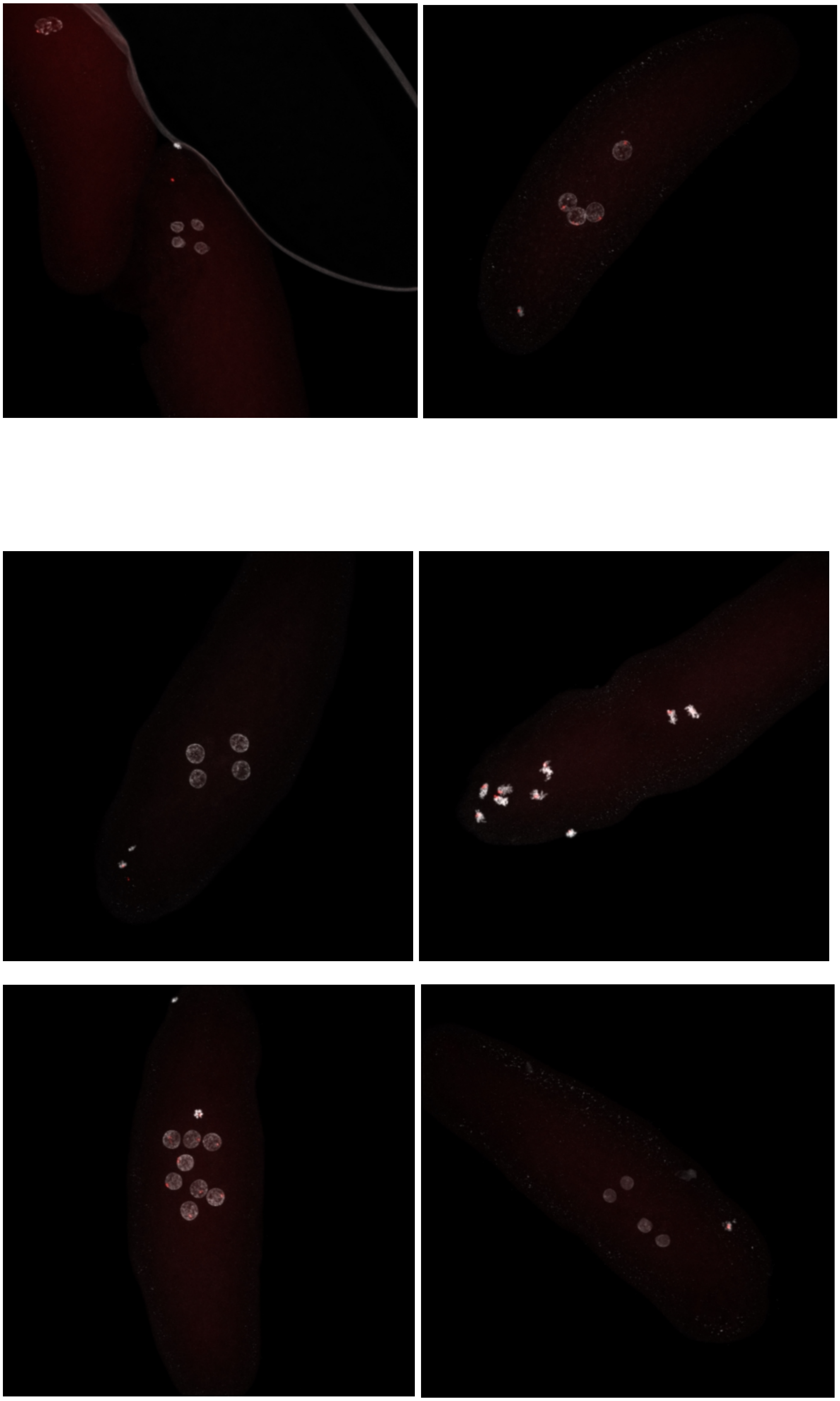

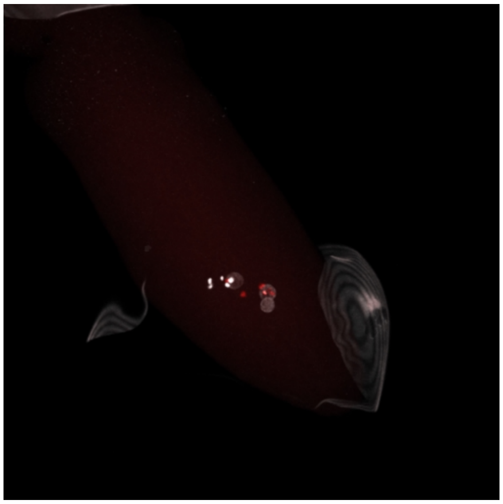
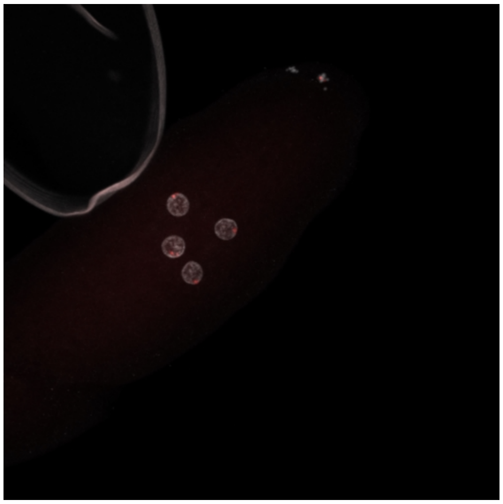
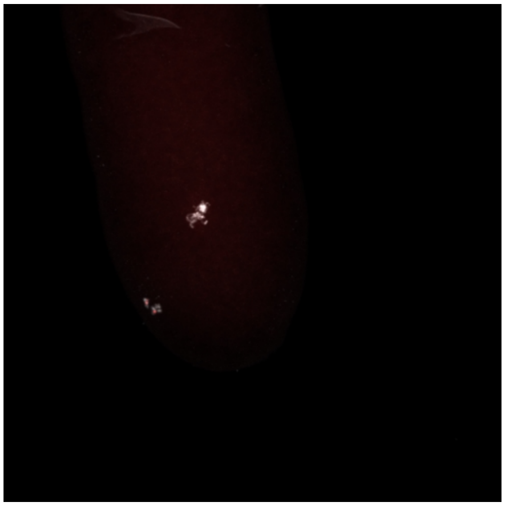
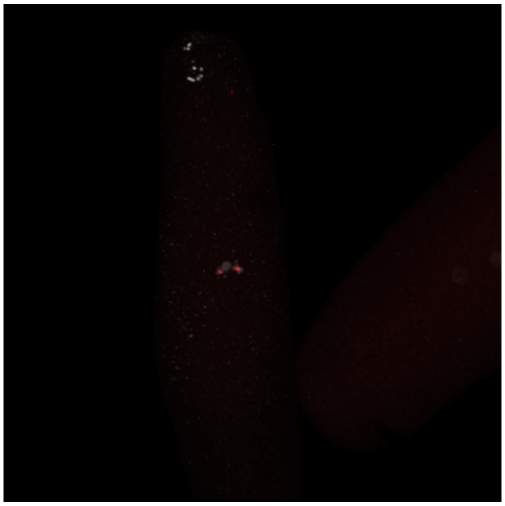

Images 001-024, taken 12-19-23 first set

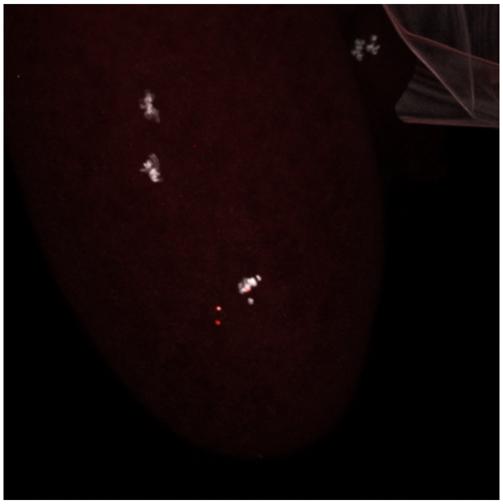

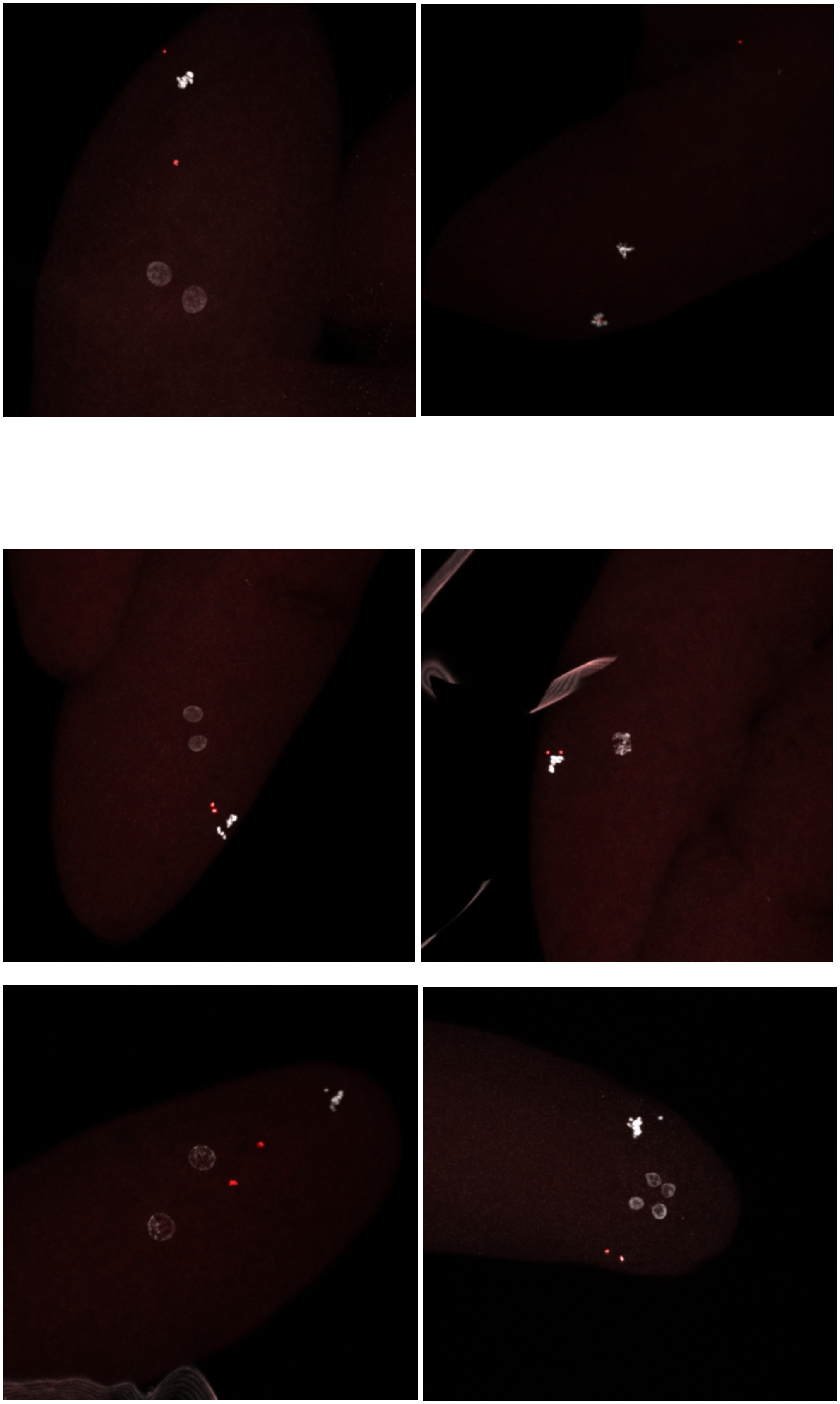

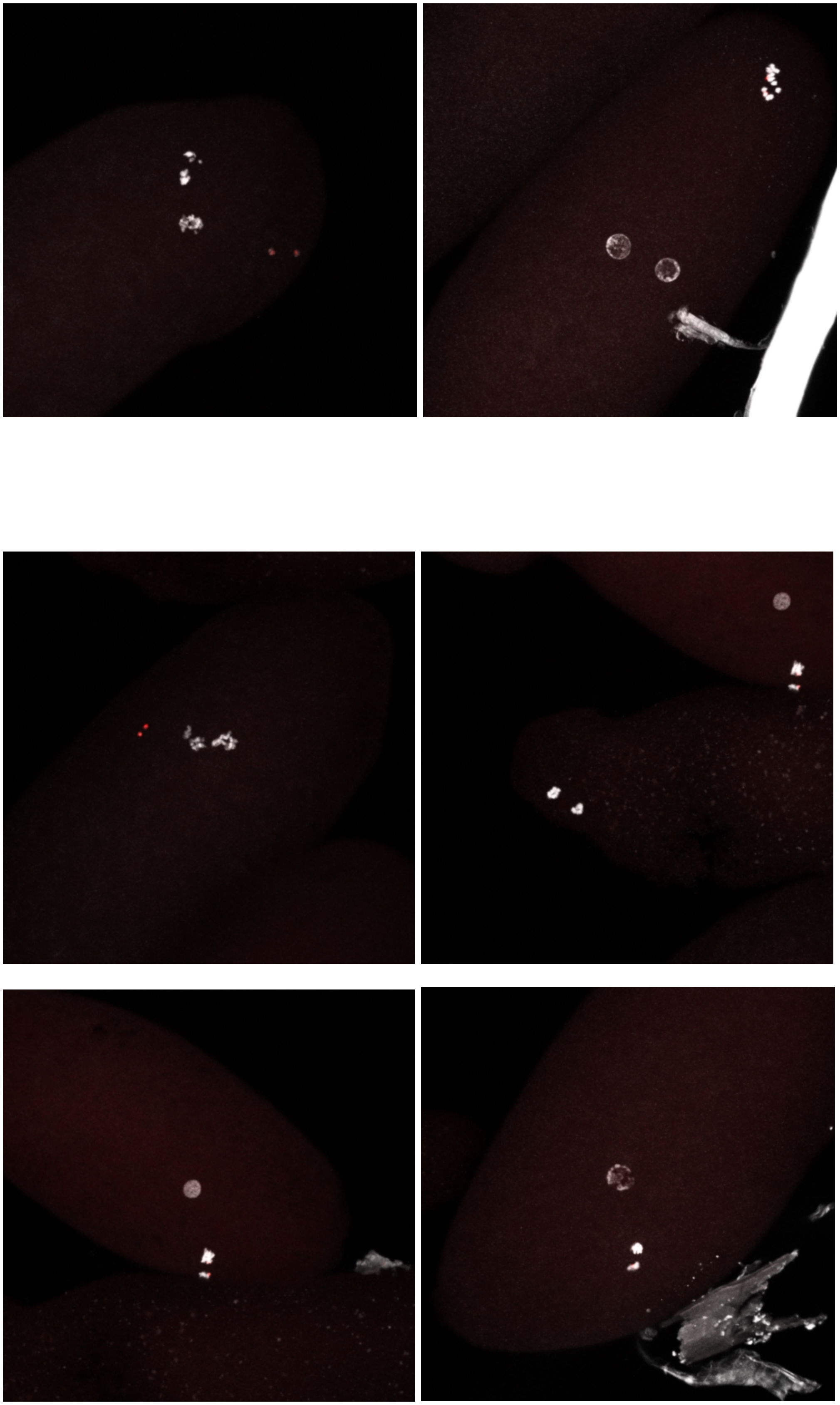

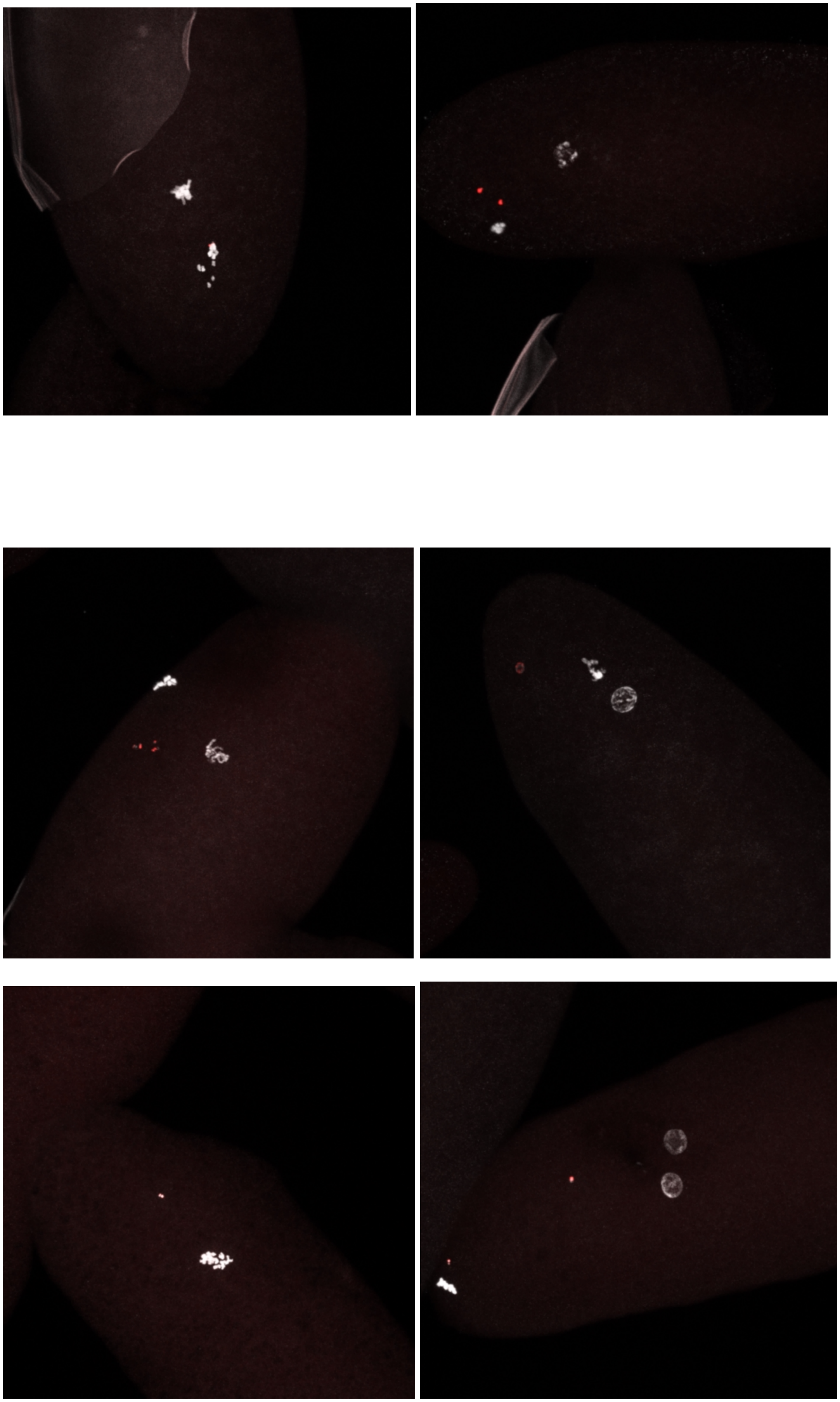

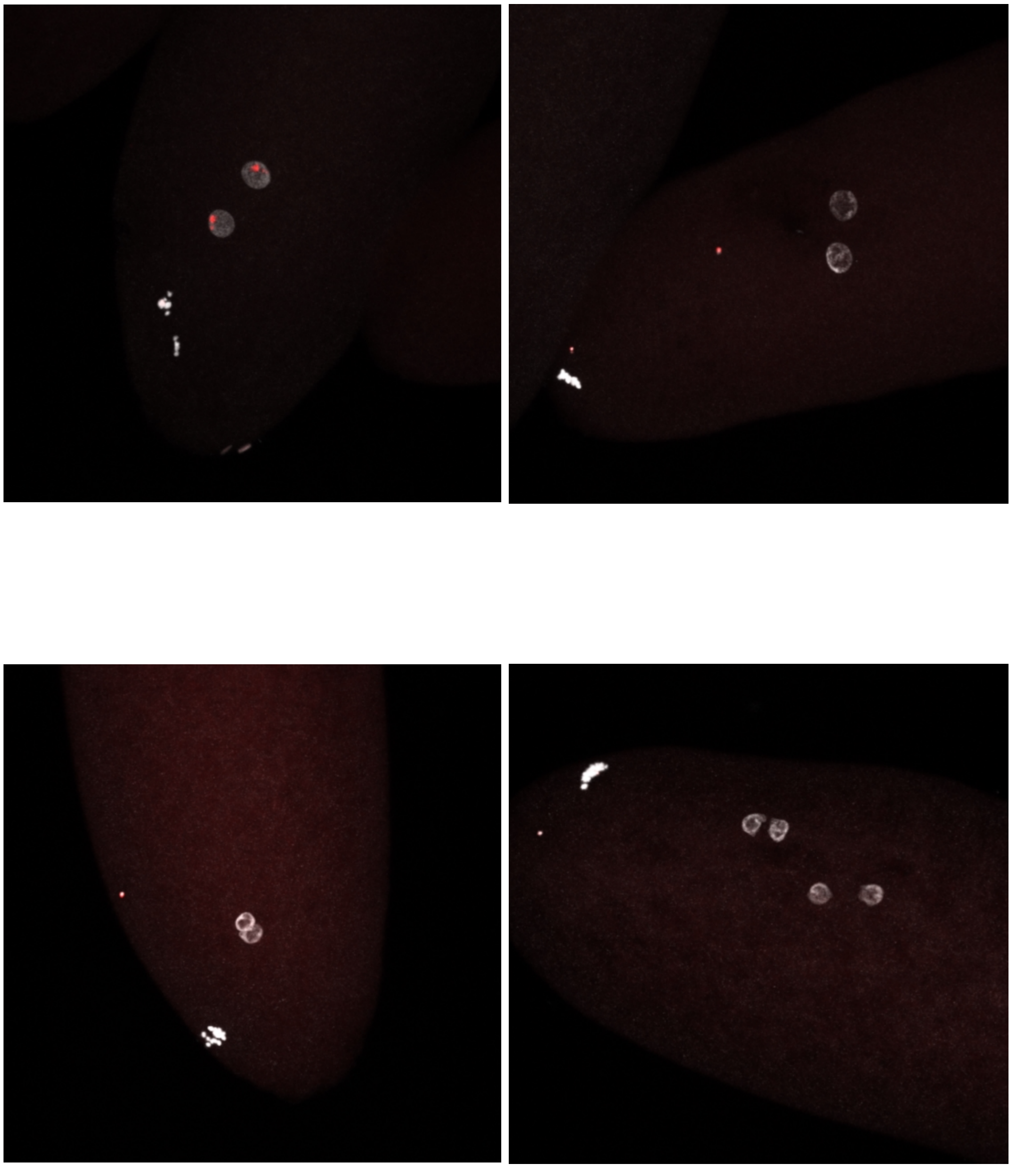

Images 001-025, taken 12-19-23 second set

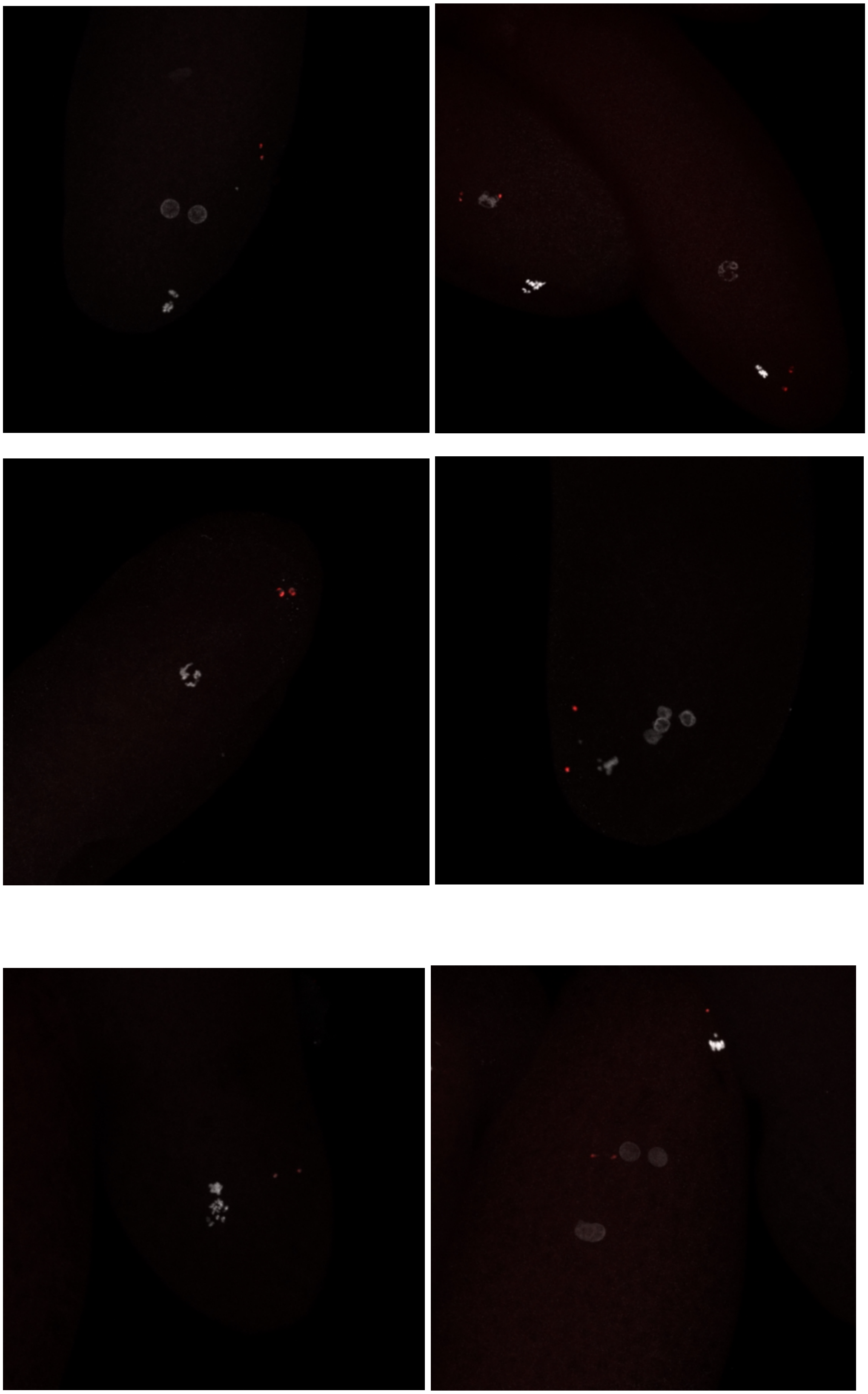

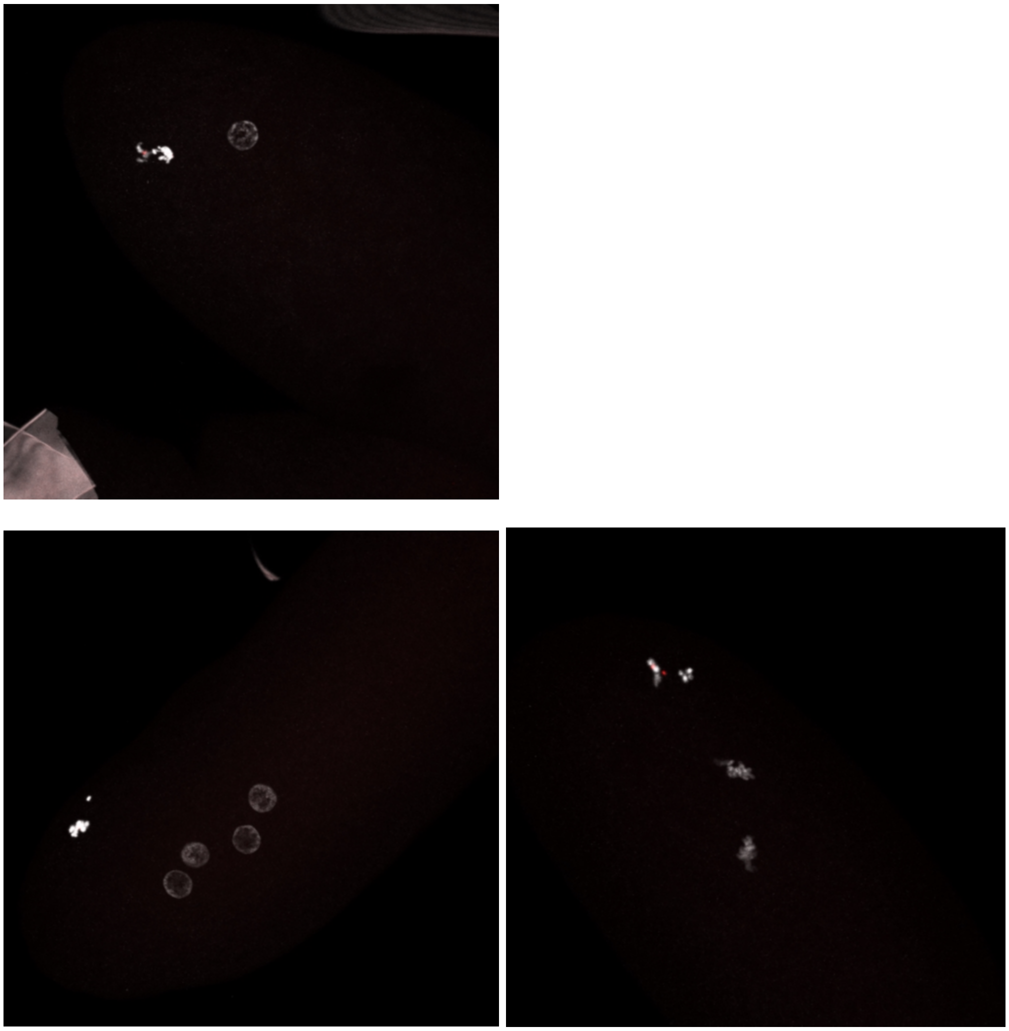

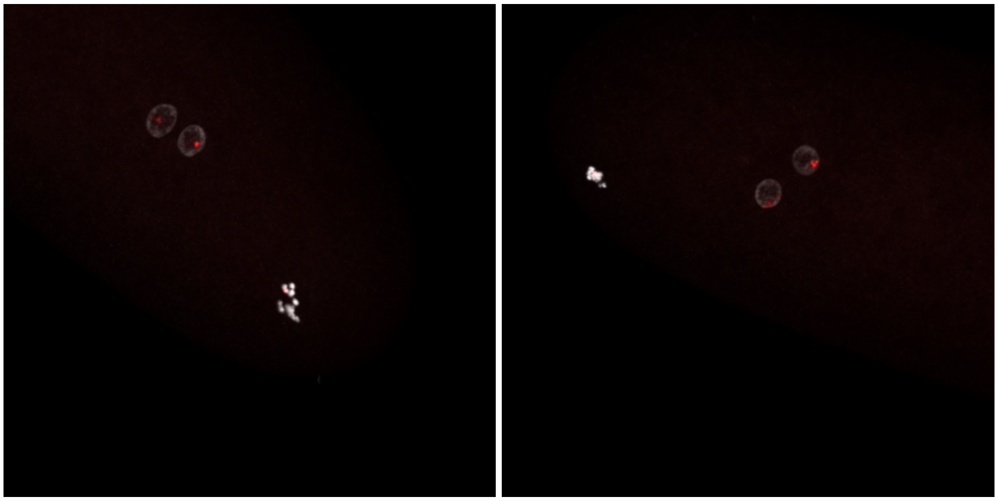

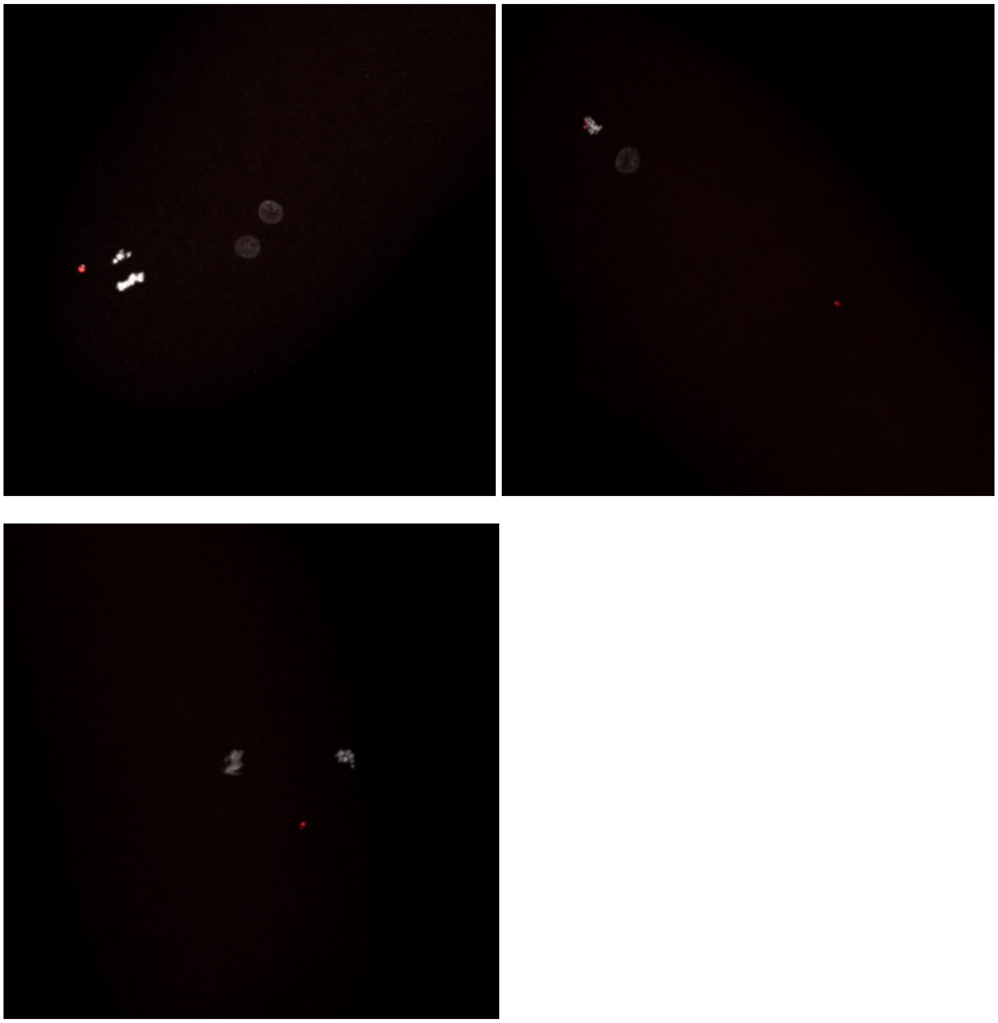

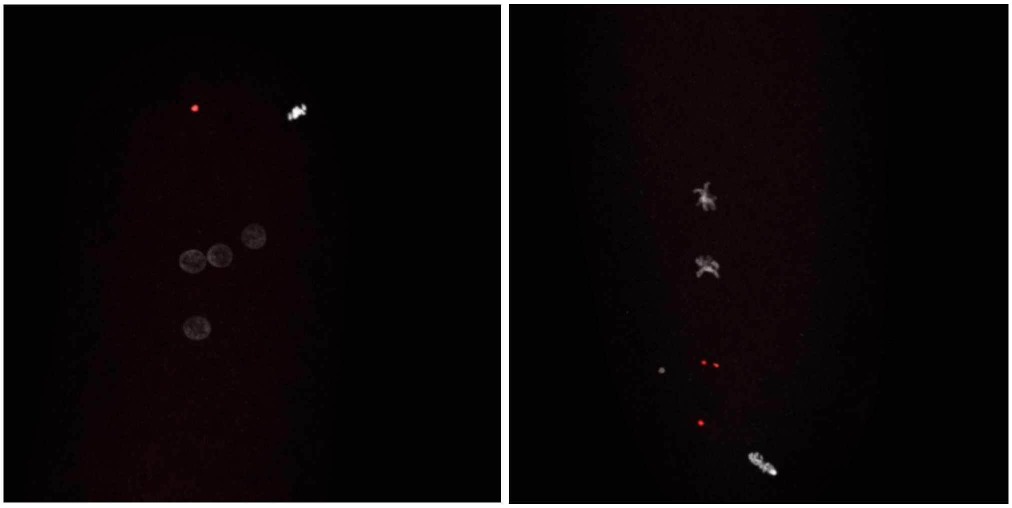

Images 001-002, taken 12-19-23 third set

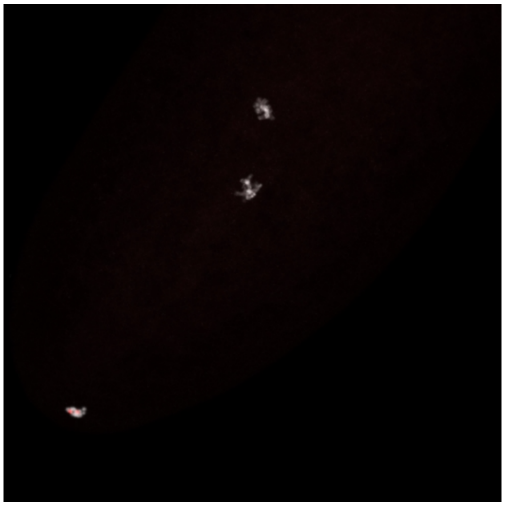
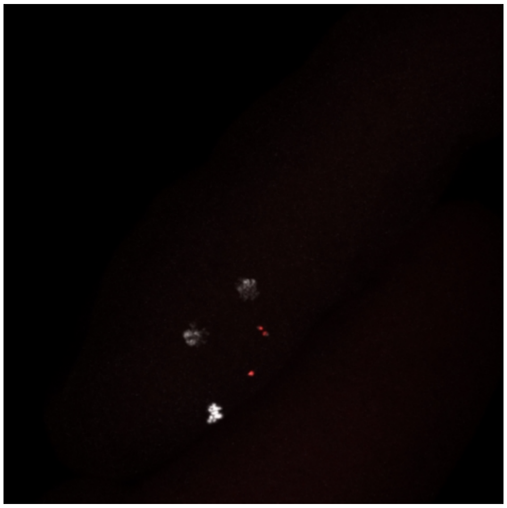
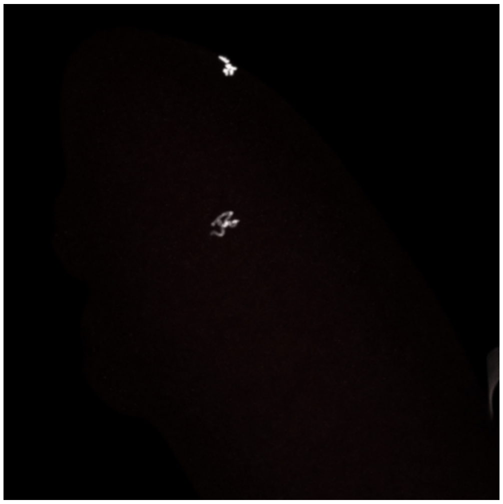
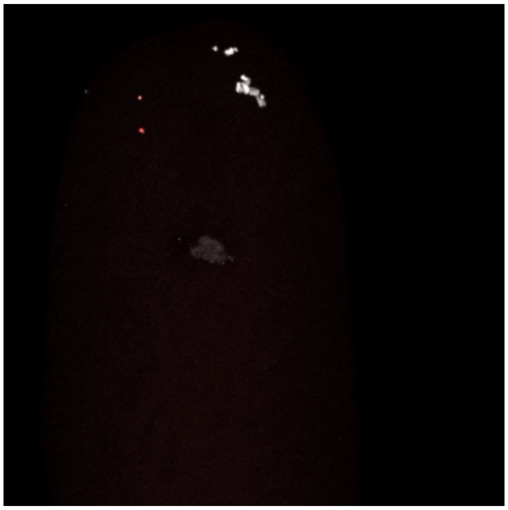

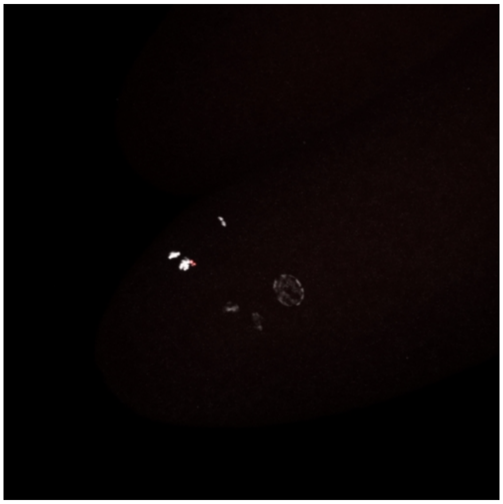
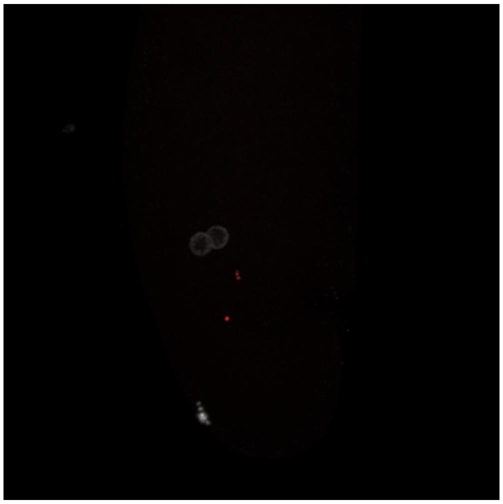

Images 001-002, taken 12-19-23 third set

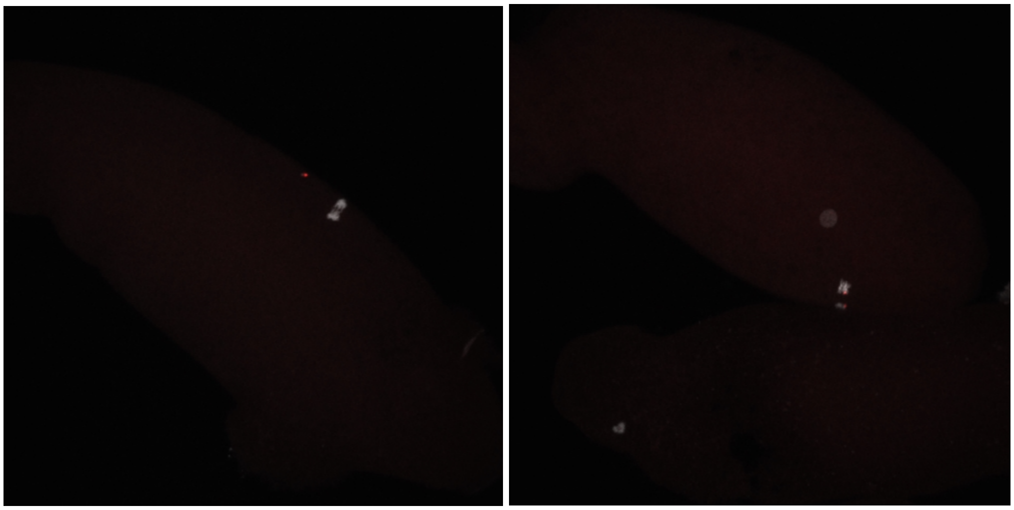

Supplement: S6 Data — PSR is red and DNA is gray. Both channels are merged. (PDF) [file pbio.3003599.s006.pdf]
