# Supplementary material for: A male-transmitted B chromosome undergoes strong meiotic drag in females of the jewel wasp Nasonia vitripennis
Source: PLoS Biol. 2026 Jan 16;24(1):e3003599. doi: 10.1371/journal.pbio.3003599 (PMC12826520; doi:10.1371/journal.pbio.3003599)

S7 Data

0-2hr embryos laid by PSR+ females mated with PSR+ males to measure PSR copy number in progeny at this early developmental stage. PSR is red and DNA is grey.

Images 012-046, taken 6-18-24

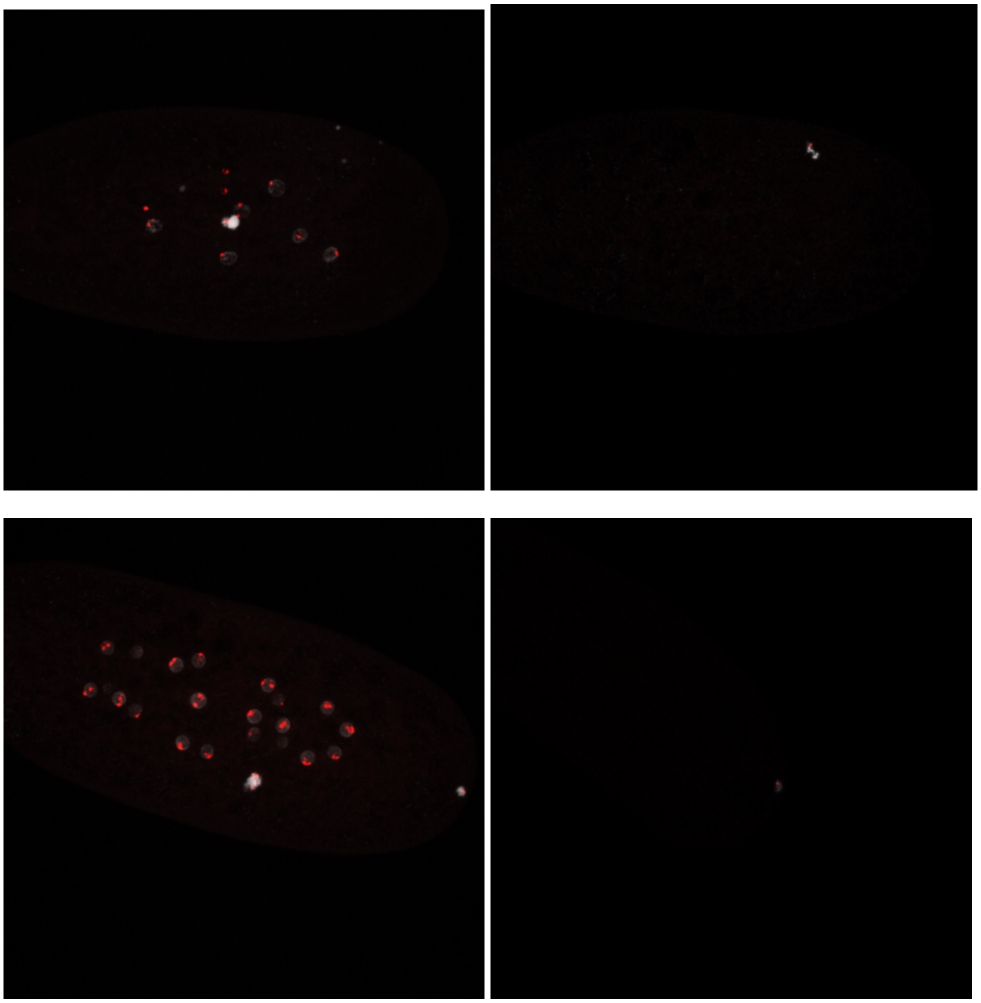

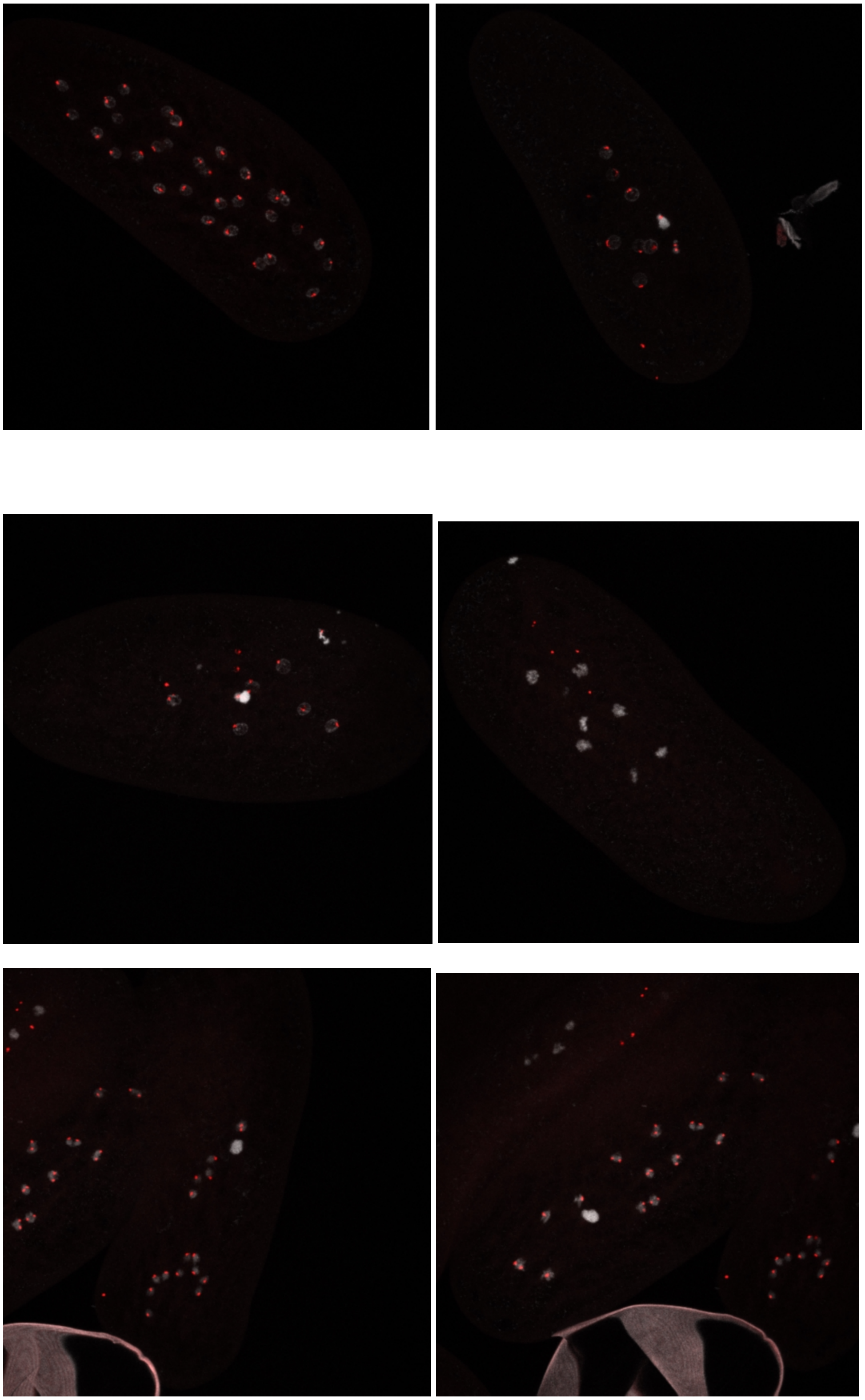

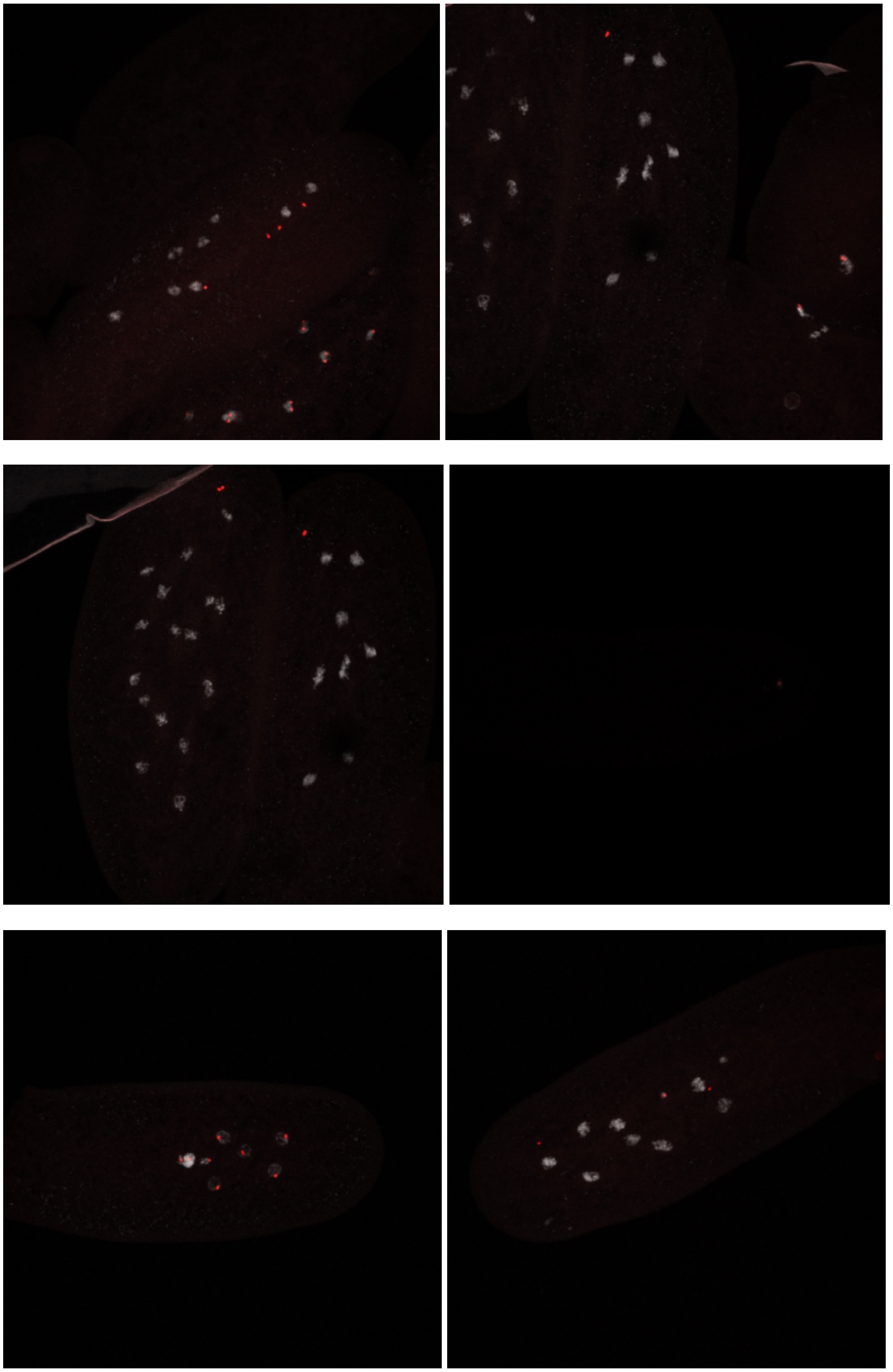

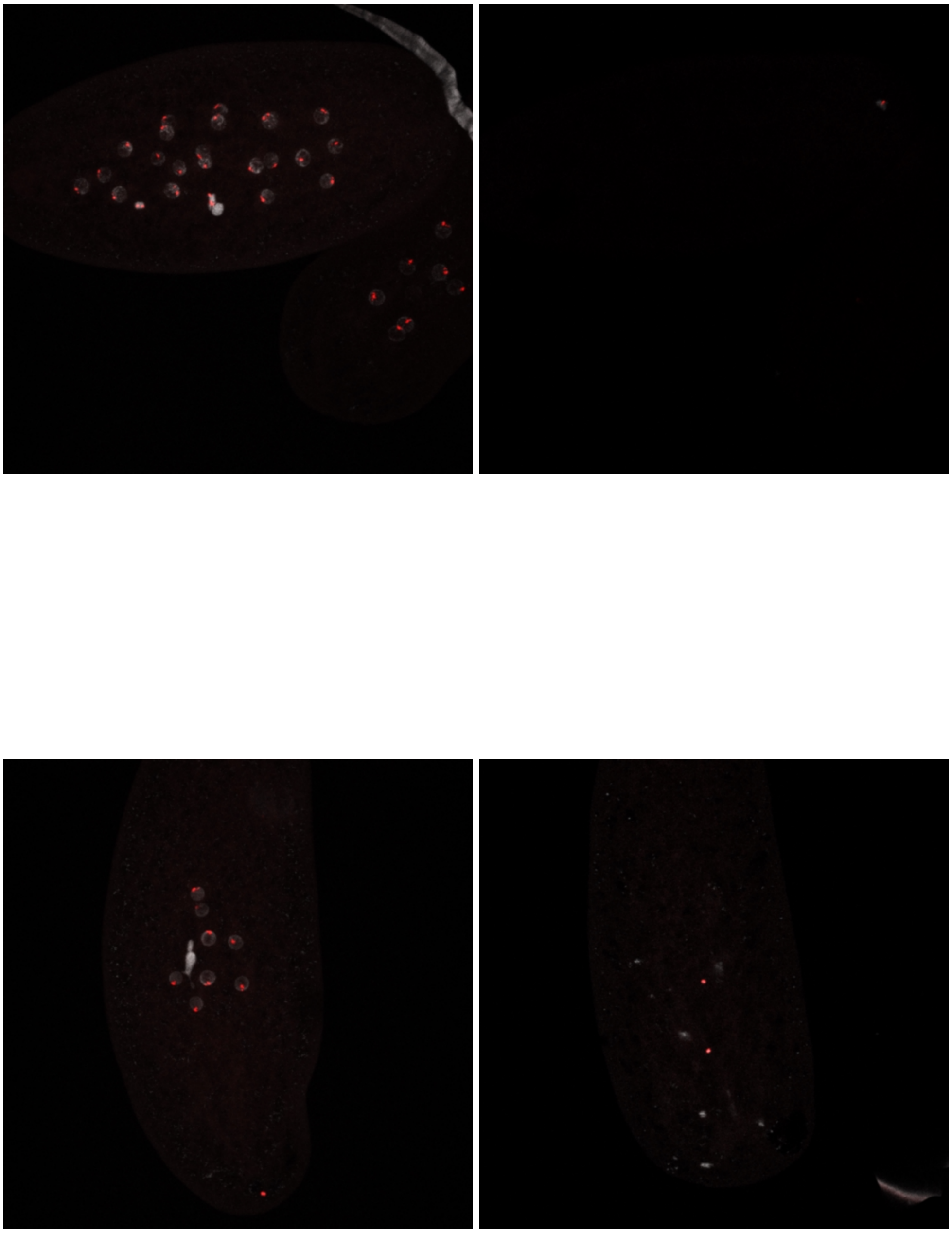

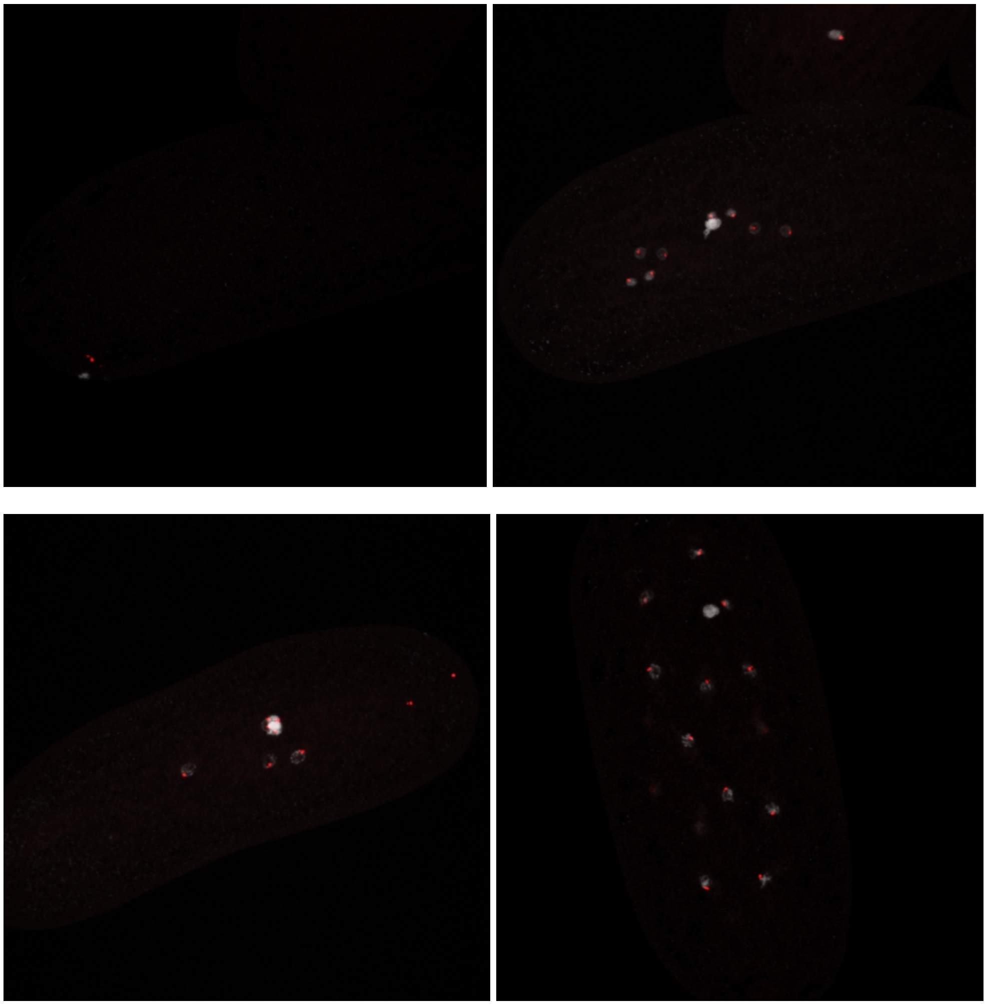

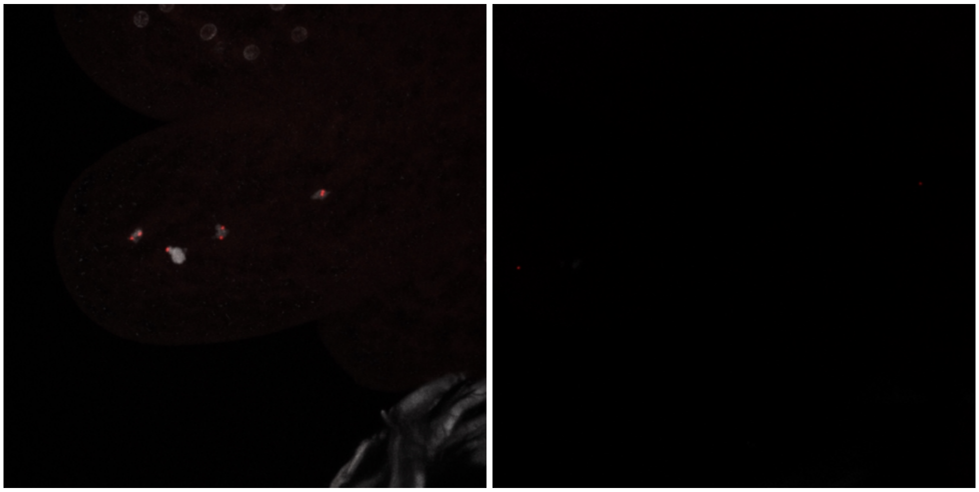

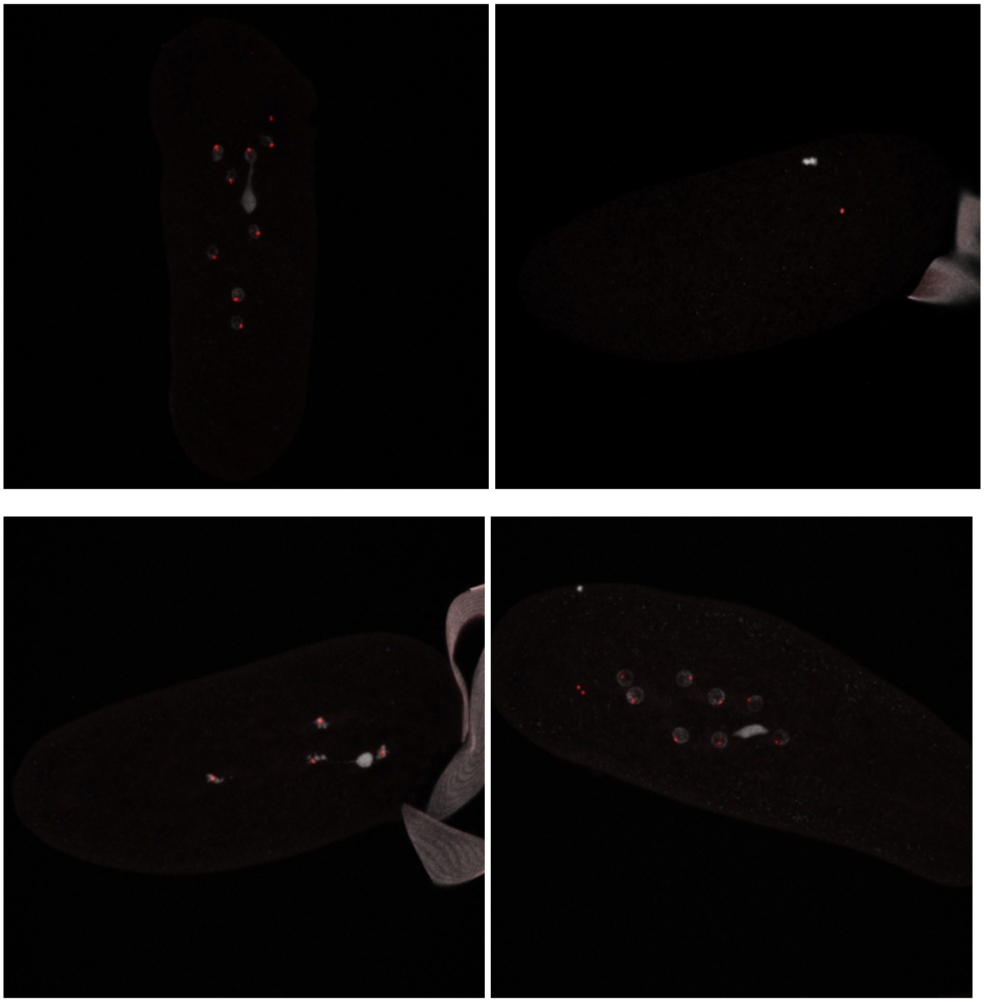

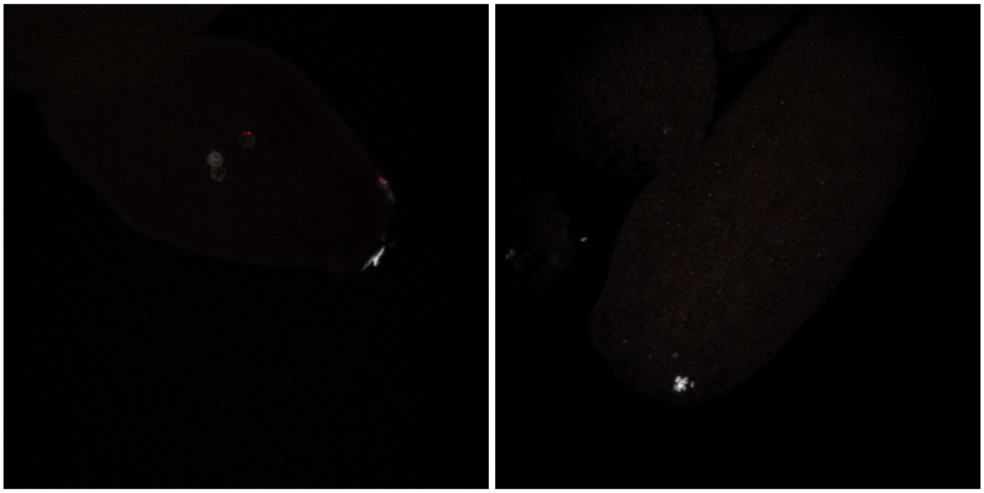

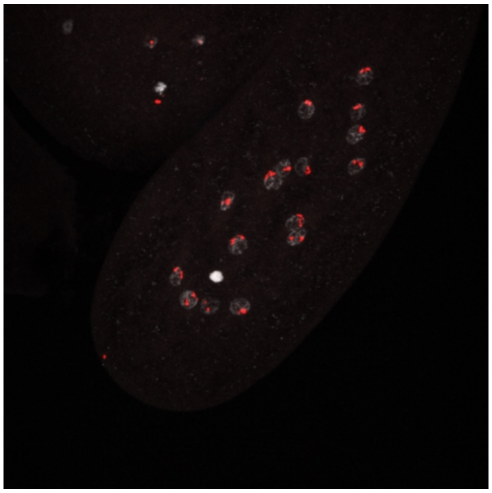

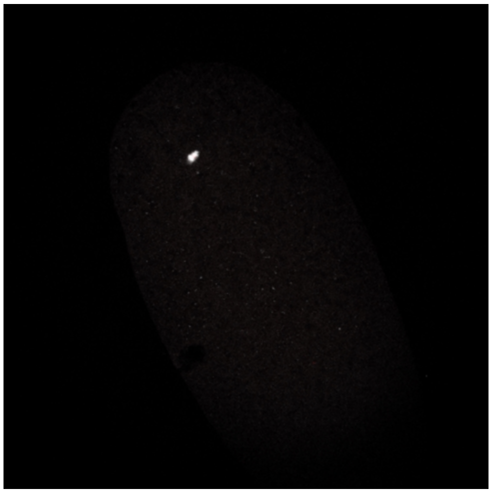

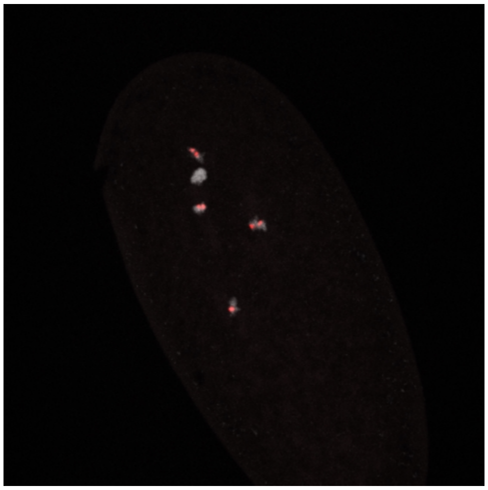

Images 001-015, taken 7-17-24

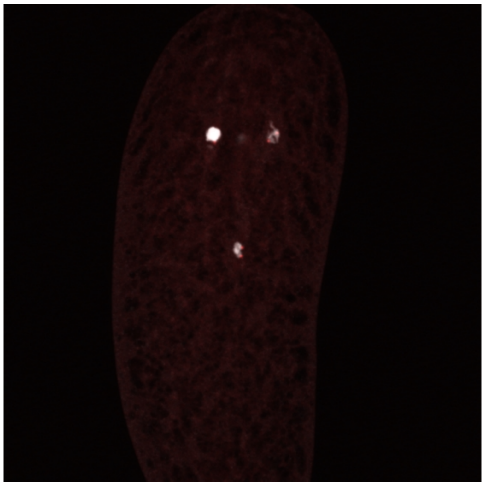

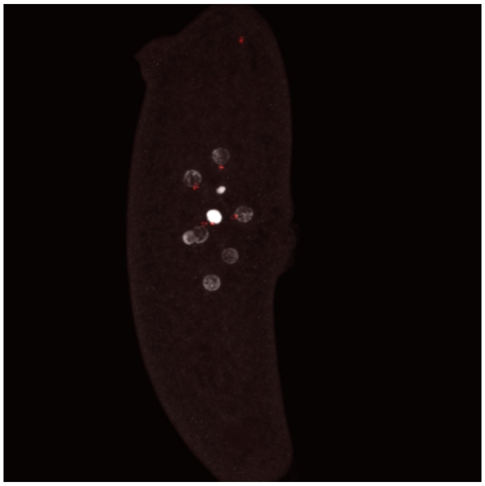

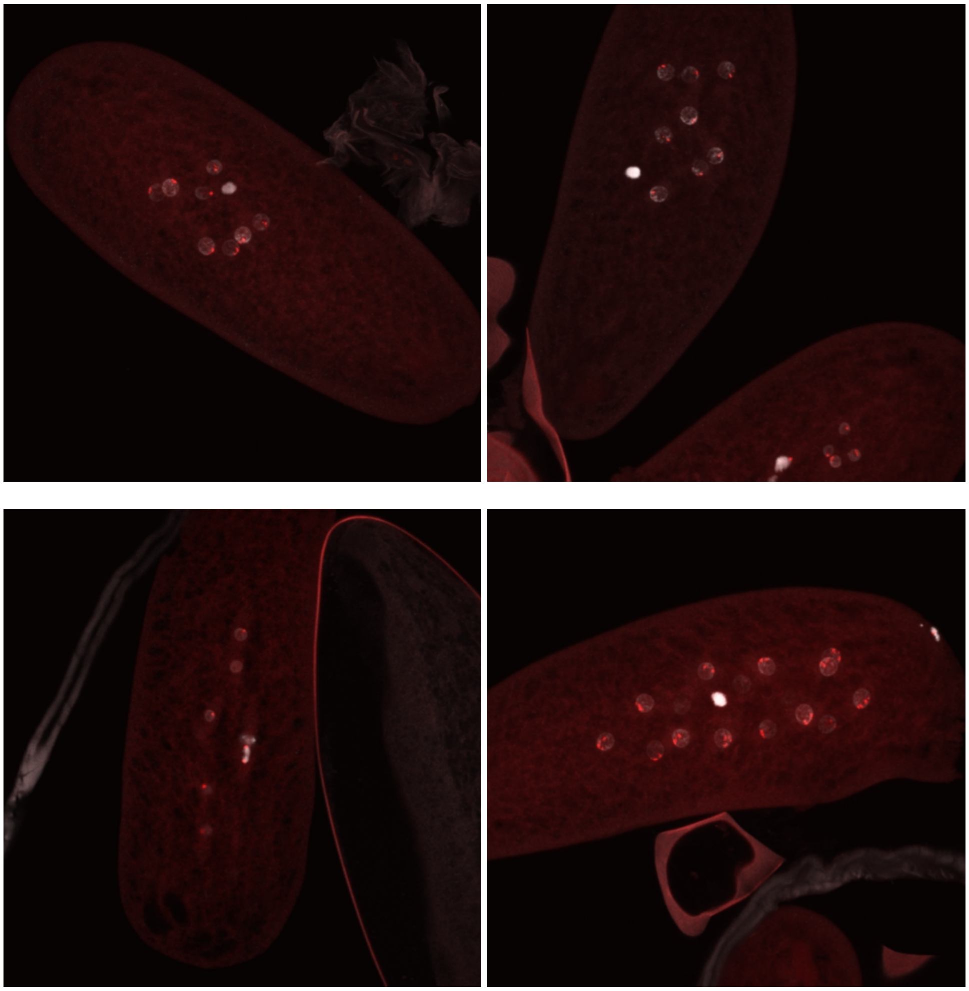

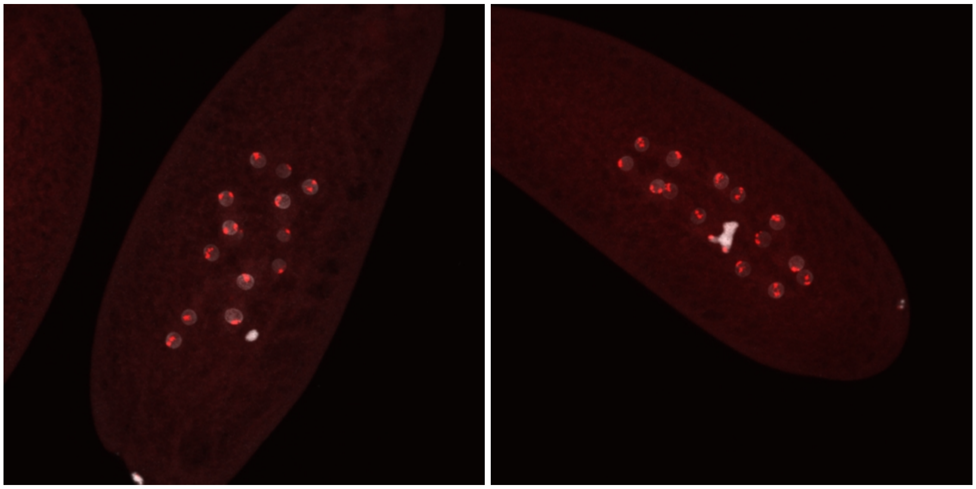

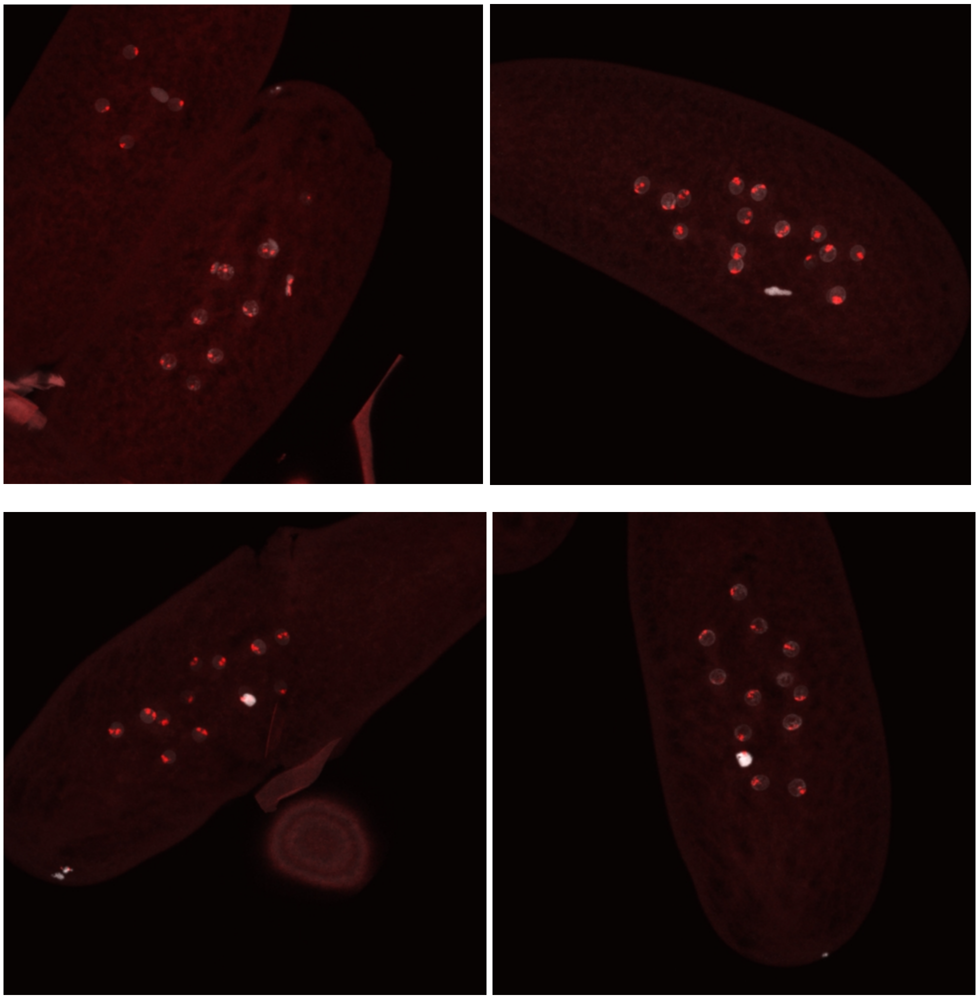

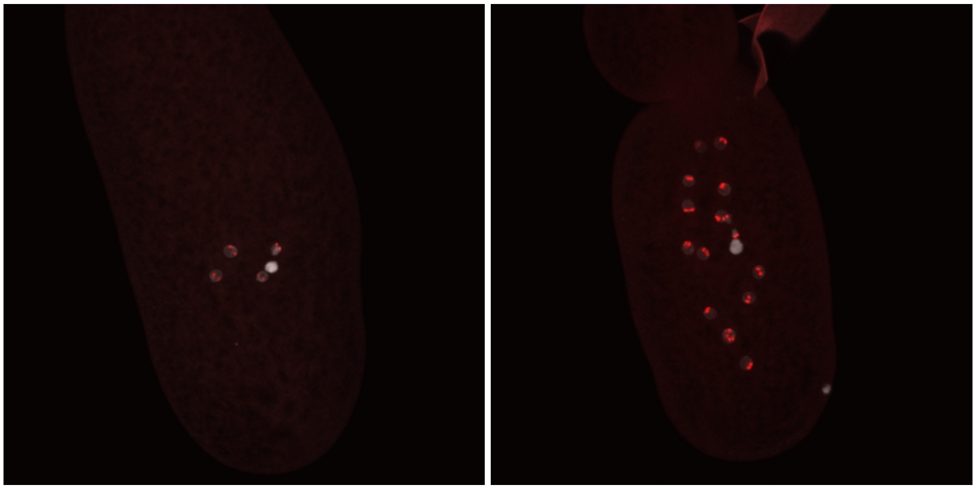

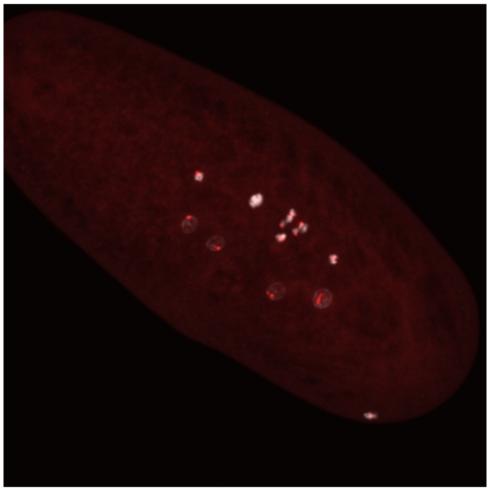

Supplement: S7 Data — PSR is red and DNA is gray. Both channels are merged. (PDF) [file pbio.3003599.s007.pdf]
